# Multiomic single nuclei profiling the mouse hippocampus reveals that ACSS2 confers neuronal resilience to tauopathy

**DOI:** 10.1002/alz.70998

**Published:** 2026-02-13

**Authors:** Gabor Egervari, Desi C. Alexander, Hua Huang, Greg Donahue, Connor Hogan, Mariel Mendoza, Hong Xu, Virginia Lee, Ben Garcia, Nancy Bonini, Shelley Berger

**Affiliations:** ^1^ Penn Epigenetics Institute Perelman School of Medicine University of Pennsylvania Philadelphia Pennsylvania USA; ^2^ Department of Cell and Developmental Biology Perelman School of Medicine University of Pennsylvania Philadelphia Pennsylvania USA; ^3^ Department of Genetics Washington University School of Medicine in St. Louis St. Louis Missouri USA; ^4^ Department of Biochemistry and Molecular Biophysics Washington University School of Medicine St. Louis Missouri USA; ^5^ Department of Pathology and Laboratory Medicine Perelman School of Medicine University of Pennsylvania Philadelphia Pennsylvania USA; ^6^ Department of Biochemistry and Biophysics Perelman School of Medicine University of Pennsylvania Philadelphia Pennsylvania USA; ^7^ Department of Biology University of Pennsylvania Philadelphia Pennsylvania USA; ^8^ Department of Genetics University of Pennsylvania Perelman School of Medicine Philadelphia Pennsylvania USA; ^9^ Department of Biology School of Arts and Sciences University of Pennsylvania Philadelphia Pennsylvania USA

**Keywords:** acetyl‐CoA synthetase 2, Alzheimer's disease, assay for transposase‐accessible chromatin using sequencing, epigenetics, histone acetylation, learning and memory, RNAseq, single‐nucleus sequencing, tauopathy

## Abstract

**INTRODUCTION:**

Epigenomic dysregulation contributes to Alzheimer's disease (AD) and related tauopathies. Acetyl‐CoA synthetase 2 (ACSS2), a nuclear‐localized metabolic enzyme in neurons, supports histone acetylation and learning‐related gene expression. We examined how ACSS2 loss affects molecular and behavioral phenotypes in a mouse model of tauopathy.

**METHODS:**

We induced tauopathy in ACSS2 knockout and control mice via injection of pathological human tau. We assessed transcriptomic, epigenomic, and behavioral changes, and tested long‐term acetate supplementation as a rescue strategy.

**RESULTS:**

ACSS2 loss worsened tau‐seeding‐related phenotypes, particularly in hippocampal pyramidal neurons and Cajal–Retzius cells. Acetate supplementation rescued learning in an ACSS2‐dependent manner and restored gene expression linked to cognition.

**DISCUSSION:**

ACSS2 acts as a neuroprotective metabolic enzyme in vulnerable hippocampal neurons, and targeting this pathway through dietary supplementation may offer therapeutic potential for AD and related tauopathies.

**Highlights:**

- We combine tau seeding with deletion of acetyl‐CoA synthetase 2 (ACSS2) to test this enzyme in an Alzheimer's disease model.
- Loss of ACSS2 exacerbates transcriptional and behavioral responses to tau injection.
- We observe robust transcriptional dysregulation in pyramidal neurons in the hippocampus.
- We observe reduced numbers of reelin‐producing Cajal–Retzius cells in the hippocampus.
- Acetate supplementation rescues transcriptional and behavioral responses to tau.

## 1 BACKGROUND

Therapeutic interventions targeting well‐established pathological aspects of Alzheimer's disease (AD), like amyloid plaques and tau phosphorylation, continue to show limited clinical sucess.^1^ Ongoing efforts to uncover additional cellular and molecular underpinnings of AD and tauopathies in general are thus of critical importance. Single‐cell and single‐nuclei technologies have identified neuronal and non‐neuronal cell populations that play key roles in AD, showing transcriptional remodeling of several key brain cell populations^2^, ^3^, ^4^, ^5^ and pointing to large‐scale, progressive epigenomic alterations as a central mechanism of cell identity loss with disease progression.^6^

Epigenetic mechanisms, particularly histone acetylation, are important in the development and progression of AD.^7^ We and others have reported altered histone acetylation in the brain of AD patients.^8^, ^9^, ^10^, ^11^ Elevation of histone acetylation improves memory across multiple mouse AD models,^12^, ^13^, ^14^, ^15^ in line with its activity‐dependent promotion of immediate early gene (IEG) expression to create synaptic circuits for memory.^16^, ^17^ Our previous analysis of *post mortem* human brains similarly found that while normal aging is characterized by increased acetylation of histone H4K16 (H4K16ac), this modification is strikingly reduced in AD.^8^ This pattern suggests that H4K16ac may have a protective effect during normal aging that is lost in AD brains. Similarly, reduction of H3K27ac in AD patient‐derived induced pluripotent stem cell neurons blocked the activation of critical homeostatic pathways, leading to enhanced cellular stress and amyloid beta (Aβ) secretion.^18^ Together, these data suggest that reduced histone acetylation exacerbates AD across multiple disease models, though the molecular mechanisms and cell‐type specificity of this process remain poorly understood.

Recent evidence for an intricate interaction between metabolic and epigenetic processes has led to a fundamental shift in models of gene regulation. In particular, metabolic enzymes have emerged as central players in epigenetic regulation.^19^, ^20^ Cytosolic and mitochondrial metabolic enzymes can translocate to the nucleus, where they generate a local supply of metabolites, including acetyl‐CoA, that serve as substrates or cofactors for histone‐ and DNA‐modifying enzymes. This metabolic–epigenetic interface provides a potential focus for novel therapeutic interventions in a variety of conditions linked to epigenetic and transcriptional dysregulation.

Here, we assess the contribution of acetyl‐CoA synthetase 2 (ACSS2), a novel metabolic regulator of brain histone acetylation,^21^, ^22^, ^23^ to epigenetic and transcriptional changes in a tau‐seeding model. In neurons, ACSS2 is predominantly nuclear,^21^ and contributes to histone acetylation and gene expression underlying hippocampal learning and memory.^21^, ^22^, ^24^ Upregulation of ACSS2 enhances synaptic gene expression in a mouse model overexpressing Aβ.^25^ However, while AD is characterized by both amyloid plaques and tau neurofibrillary tangles, tau‐associated dysfunction is more directly linked to disease progression.^26^, ^27^, ^28^ Several models for tau toxicity and pathology have been developed in the mouse by injecting human AD‐associated tau aggregates into the mouse brain.^29^ These models show pathology that mimics human disease, such as spreading of tau aggregates, and thus are useful models to capture pathological aspects of human tauopathies in mice.

In this study, we used human pathological AD tau to seed the hippocampus of constitutive ACSS2 knockout (ACSS2 KO) mice to query long‐term, steady‐state cellular and genomic outcomes associated with the loss of ACSS2 in the context of tauopathy. Importantly, the combination of developmental loss of ACSS2 and chronic incubation with AD tau (6 months post‐injection) in this model enabled characterization of complex cellular and molecular outcomes in the brain encompassing both direct and indirect effects of ACSS2 loss. Such an approach is critical to establish the key neuroprotective role of ACSS2 in the context of tauopathy and related cognitive impairments.

We find that molecular changes after hippocampal AD tau seeding in part resemble those observed in human AD, and, in the context of ACSS2 KO, tau injection leads to a loss of neuroprotective gene expression programs and a striking gain of risk‐conferring pathways that result in loss of hippocampal cell identity. We define ACSS2 as a key modulator of AD tau–related molecular and behavioral phenotype. Our findings point to therapeutically targetable mechanisms underlying the onset and progression of tauopathy that are independent of traditional efforts focused on tau accumulation.

## 2 METHODS

### 2.1 Mice

Animal use and all experiments performed were approved by the institutional animal care and use committee (IACUC, protocols 804849). All personnel involved have been adequately trained and are qualified according to the Animal Welfare Act (AWA) and the Public Health Service (PHS) policy. To prevent genetic drift, Acss2^KO^ and corresponding wild‐type (WT) controls were generated through homozygous crosses of F1 progeny from heterozygote breeding cages. Mice were housed on a 12/12 hour light/dark cycle (7:00 am to 7:00 pm), with food and water provided ad libitum. To investigate the effects of chronic acetate treatment, mice were maintained on LabDiet 5001 with 5% Na‐acetate or a calorically equivalent control (manufactured and irradiated by TestDiet, Land O'Lakes Inc.). All behavioral experiments were conducted between 7:00 am and 11:00 am to reduce time‐of‐day effects. Female mice were used for all behavioral and single‐cell experiments. Male mice were used for bulk RNA sequencing (RNA‐seq) and chromatin immunoprecipitation sequencing (ChIP‐seq).

### 2.2 Purification of pathological tau protein from human AD brains

Tau protein was purified as previously described.^29^ Briefly, 6 to 14 g of frontal cortical gray matter from AD patients was homogenized using a Dounce homogenizer in nine volumes (v/w) of high‐salt buffer (10 mM Tris‐HCl, pH 7.4, 0.8 M NaCl, 1 mM ethylenediaminetetraactic acid [EDTA], and 2 mM dithiothreitol [DTT], with protease inhibitor cocktail, phosphatase inhibitor, and phenylmethylsulfonyl fluoride), with 0.1% sarkosyl and 10% sucrose added and centrifuged at 10,000 × g for 10 minutes at 4°C. Pellets were re‐extracted once or twice using the same buffer conditions as the starting materials, and the supernatants from all two to three initial extractions were filtered and pooled. Additional sarkosyl was added to the pooled low‐speed supernatant to reach 1%. After 1 hour of nutation at room temperature, samples were centrifuged again at 300,000 × g for 60 minutes at 4°C. The resulting sarkosyl‐insoluble pellets, which contain pathological tau, were washed once in phosphate‐buffered saline (PBS) and then resuspended in PBS (≈ 100 µL/g gray matter) by passing through 27‐G 0.5 inch needles. The resuspended pellets were further purified by a brief sonication (20 pulses at ≈ 0.5 seconds/pulse) using a hand‐held probe (QSonica), followed by centrifugation at 100,000 × g for 30 minutes at 4°C, whereby the majority of protein contaminants were partitioned into the supernatant, with 60% to 70% of tau remaining in the pellet fraction. The pellets were resuspended in PBS at one fifth to one half of the pre‐centrifugation volume, sonicated with 20 to 60 short pulses (≈ 0.5 seconds/pulse), and spun at 10,000 × g for 30 minutes at 4°C to remove large debris. The final supernatants were used in the study and referred to as AD tau. The use of *post mortem* brain tissues for research was approved by the University of Pennsylvania's Institutional Review Board with informed consent from patients or their families.

**RESEARCH IN CONTEXT**

1. **Systematic review**: Previous work identified acetyl‐CoA synthetase 2 (ACSS2), a metabolic enzyme localized to neuronal nuclei, as a key regulator of gene expression underlying learning and memory, suggesting that this pathway may be similarly important in neurodegenerative (tauopathy‐related) memory loss.
2. **Interpretation**: While loss of ACSS2 is generally well tolerated under homeostatic conditions, the dual insult of ACSS2 loss and tau aggregation results in substantial transcriptional dysregulation along hippocampal circuits and loss of Cajal–Retzius neurons. However, dietary supplementation with acetate ameliorates tau‐related memory decline and reverses gene expression changes, suggesting potential dietary therapies for human patients.
3. **Future directions**: The role of metabolites and their impact on the epigenome of the brain remain understudied in health and disease. While this and other work suggests that metabolic pathways can be leveraged to improve neuronal resilience in mice, further studies, especially in human populations, are necessary.

### 2.3 AD tau injections

Six‐month‐old male and female WT and ACSS2 KO mice were anaesthetized with isoflurane gas (1%–5% to maintain surgical plane) and placed in a sterile field within a stereotaxic device. Artificial tears were applied to eyes to ensure sufficient lubrication. Animals received an injection of bupivacaine (2.5 mg kg^−1^) for local anesthesia before the skin was disinfected with betadine solution and the skull exposed with a short incision using sterile surgical equipment. A small hole (≈ 0.5 mm) was drilled in the skull over the target area using a stereotaxic and a stereotactic drill. A micro‐syringe filled with AD tau lysate was inserted into the right dorsal hippocampus and slowly removed after injection of 1 µg tau (AP −2.5 mm; DV −1.4 mm; ML +2 mm from bregma). All animals received a single dose of subcutaneous meloxicam (5 mg/kg) as analgesia at induction and one dose per day for 2 days postoperatively as needed. To allow sufficient time for the development of AD‐like pathology, mice underwent behavioral or molecular characterization 6 months post‐injection.

### 2.4 Fear conditioning

Mice were handled for 3 consecutive days for 2 minutes each prior to the start of the experiment. On the day of fear acquisition, mice were individually placed in conditioning chambers (Med Associates) and habituated to the novel environment for 2.5 minutes prior to delivery of a 2 second long, 1.5 mA foot shock. Mice were promptly removed 30 seconds after shock onset and returned to their home cages. Chambers were wiped down with 70% ethanol between each round. Twenty‐four hours later, freezing response was tested for 5 consecutive minutes using FreezeScan software (CleverSys, Inc.).

### 2.5 Object location memory

Non‐aversive spatial memory was assessed as previously described.^22^, ^30^ Briefly, mice were handled for 2 minutes a day for 3 consecutive days (KO). On training day, mice were habituated to an empty arena for 6 minutes, then exposed to a collection of three distinct objects (glass bottle, metal tower, and plastic cylinder) of roughly similar sizes for 6 minutes. This training was repeated three times, with mice removed and all surfaces wiped down with 70% ethanol between trainings. For testing, the mice were returned to the same arena 24 hours later, this time with one object moved to a new location. Mice were allowed to explore freely for 6 minutes. Mice were monitored using a video camera, and time spent interacting with each object was assessed afterward using Any‐maze (Stoelting Co.). Quantification of mouse–object interactions was blinded. Discrimination index was calculated according to

$$\begin{array}{ccc} \text{DI} & = & (t_{\mathrm{displaced}}-\left[\left(t_{\mathrm{stat}\mathrm{iona}\mathrm{ry}1}+t_{\mathrm{stat}\mathrm{iona}\mathrm{ry}2}\right)/2\right])/\\ & & (t_{\mathrm{displaced}}+\left[\left(t_{\mathrm{stat}\mathrm{iona}\mathrm{ry}1}+t_{\mathrm{stat}\mathrm{iona}\mathrm{ry}2}\right)/2\right])\times 100 \end{array}$$

### 2.6 Open field

Mice were placed in an open arena (30 cm × 40 cm) and allowed to explore freely for 6 minutes. Resulting videos were analyzed using ANY‐maze behavioral tracking software (Stoelting Co.). Locomotion was assayed using path length (in meters) over the entire 6 minute period. Thigmotaxis (a measure of anxiety), was measured by quantifying the amount of time spent within the peripheral zone (defined as 8 cm from the arena walls) over the entire period.

### 2.7 Elevated zero maze

The maze was a circle‐shaped platform of 5 cm in diameter with two opposing open arcs and two opposing closed arcs of equal size, elevated 30 cm above the ground.^31^ Mice were placed in the middle of a closed arc and were allowed to freely explore the maze for 5 minutes. Each test was videotaped, and the behavior of the mouse was subsequently scored by an independent and blinded observer. Arm entry was defined as entering an arm with all four paws. At the end of the 5 minute test period, the mice were removed from the maze, the floor was wiped with a damp cloth, and any fecal boluses removed. The percentage of time spent in the open arcs (time spent in open arcs/[time spent in open arcs + time spent in closed arcs] × 100) and the percentage of the number of open‐arc entries (open arcs entries/[open arcs entries + closed arcs entries] × 100) were used as a measure of anxiety.

### 2.8 Y‐maze

The Y‐maze test of spontaneous alternation assess working spatial memory in rodents.^32^ Mice were gently placed in the distal portion of an arm in a Y‐shaped arena and allowed to explore freely. Mice were recorded by video cameras, and arm entries were scored by eye by blinded investigators. Arm entries were called when the mouse's hindquarters passed fully into the new arm. Percent spontaneous alternations were quantified according to the formula (SAR = [Number of alternations/(total arm entries – 2)] × 100).

### 2.9 Histology and immunohistochemistry

Animals were transcardially perfused with 1× PBS followed by 4% paraformaldehyde in PBS. After dissection, brain tissues were transferred to PBS and subsequently embedded in paraffin and sectioned at a thickness of 5 µm. Every tenth section was stained with hematoxylin and eosin (H&E), and sections from similar anatomical planes were chosen for histologic and immunohistochemical analyses. After deparaffinization and rehydration, sections were stained with phosphor‐Tau (Ser202, Thr205) monoclonal antibody clone AT8 (Thermo, Cat. #MN1020) to detect tau phosphorylation. Staining was quantified using QuPath software within manually curated regions of interest (ROIs) and using an automated threshold‐based classifier. The classifier was consistently applied across all samples and conditions. Histopathologic analysis was performed in a blinded manner.

### 2.10 Histone extraction, propionylation, and digestion

Histones were extracted from 1 mm^2^ punches taken from the dorsal CA1 region of the mouse hippocampus by using nuclei isolation buffer (NIB), as previously described.^33^, ^34^ The tissue punches were homogenized using biovortexer in NIB (15 mM Tris‐HCl, 15 mM NaCl, 60 mM KCl, 5 mM MgCl_2_, 1 mM CaCl_2_ and 250 mM sucrose at pH 7.5; 0.5 mM 4‐[2‐aminoethyl]benzenesulfonyl fluoride hydrochloride, 10 mM sodium butyrate, 5 µM microcystin, and 1 mM DTT added fresh) with 0.3% NP‐40 on ice, and washed twice in NIB without NP‐40 to remove the detergent from the nuclear pellet. Next, pellets were incubated in 0.2 M H_2_SO_4_ for 4 hours, and the supernatant was collected after centrifugation for 5 minutes at 3400 × g. Finally, histones were precipitated with 25% trichloroacetic acid (TCA) overnight. The histone pellet was then washed with ice‐cold acetone‐0.1% HCl and acetone twice to remove residual TCA. Histones were derivatized and digested as previously described.^30^ Histone pellets were resuspended in 30 µL of 50 mM ammonium bicarbonate (pH 8.0), and 15 µL derivatization mix was added to the samples, which consist of propionic anhydride and acetonitrile in a 1:3 ratio (v/v), and this was immediately followed by the addition of 7.5 µL ammonium hydroxide to maintain pH 8.0. The sample was incubated for 15 minutes at 37 °C, dried, then the derivatization procedure was repeated one more time to ensure complete derivatization of unmodified and monomethylated lysine residues. Samples were then resuspended in 50 µL of 50 mM ammonium bicarbonate and incubated with trypsin (enzyme:sample ratio of 1:20) overnight at room temperature. After digestion, the derivatization reaction was performed again twice to derivatize the N‐termini of the peptides. Samples were desalted using C18 stage tips before liquid chromatography mass spectrometry (LC‐MS) analysis and dried. Finally, the peptides were resuspended in 0.1% formic acid (FA) prior to nano‐flow LC tandem MS (nLC‐MS/MS).

### 2.11 Mass spectrometry

Samples were analyzed by using a nLC‐MS/MS setup. NLC was configured with a 75 µm ID × 25 cm Reprosil‐Pur C18‐AQ (3 µm; Dr. Maisch GmbH, Germany) nano‐column using an EASY‐nLC nano‐HPLC (Thermo Scientific), packed in house. The high‐performance LC gradient was as follows: 5% to 32% solvent B (A = 0.1% formic acid; B = 80% acetonitrile, 0.1% formic acid) over 45 minutes, from 32% to 90% solvent B in 5 minutes, 90% B for 10 minutes, at a flow rate of 300 nL/minutes. n = LC was coupled to an Orbitrap Elite mass spectrometer (Thermo Scientific). The acquisition method was data independent acquisition (DIA) as described. Briefly, two full‐scan MS spectra (m/z 300–1100) were acquired in the ion trap within a DIA duty cycle, and 16 MS/MS were performed with an isolation window of 50 Da. Normalized collision energy (CE) was set to 35%. Raw MS data were analyzed manually. We selected the seven most intense peptides of histone H3 and H4 containing acetylations, and we extracted the relative abundance of the M+1, M+2, and M+3 isotopes compared to the monoisotopic signal. The other peptides were not considered because, due to their low abundance, we could not reliably quantify the relative abundance of all the isotopes. The percentage represented in the radar plots indicates the relative intensity of the M+3 signal (the fourth isotope) compared to the monoisotopic signal. Data were not normalized to the non‐labeled sample, so that the relative abundance of the natural isotopic distribution can be appreciated also in the untreated mice.

### 2.12 RNA extraction and RNA‐seq

RNA was extracted and libraries were prepared as previously described.^21^ Briefly, total RNA was extracted using Trizol‐chloroform from 1 mm‐thick slices of dorsal hippocampus (comprising the CA1, CA2, CA3, and dentate gyrus). Total RNA quality was assessed on the Bioanalyzer platform using the RNA 6000 Nano assay (Agilent). mRNA was isolated from 300 ng total RNA using the NEBNext Poly(A) mRNA Magnetic Isolation Module (E7490L), and libraries were prepared using the NEBNext Ultra II RNA Library Prep Kit for Illumina (E7770). All RNA‐seq data were prepared for analysis as follows: NextSeq sequencing data was demultiplexed using native applications on BaseSpace. Demultiplexed FASTQs were aligned by RNA‐STAR 2.5.2 to assembly mm10 (GRCm38; parameters: –outFilterType BySJout –outFilterMultimapNmax 20 –alignSJoverhangMin 8 –alignSJDBoverhangMin 1 –outFilterMismatchNmax 999 –alignIntronMin 20 –alignIntronMax 1000000 –alignMatesGapMax 1000000). Aligned reads were mapped to genomic features using HTSeq 0.9.1 (parameters: ‐r pos ‐s rev ‐t exon ‐i gene_id). Quantification, library size adjustment, and differential gene expression analysis were performed using DESeq2. The significance of gene alterations was determined using the Wald test with multiple test correction according to the Benjamini–Hochberg method with false discovery rate (FDR) < 0.05. Gene Ontology (GO) analysis was performed using the DAVID bioinformatics suite,^35^ and top terms associated with biological processes (FDR < 0.1) were reported. RNA‐seq tracks were created using deepTools bamCoverage v3.4.3, first splitting by tag orientation to the genomic reference strand and then creating coverage maps. Because a reads per million mapped reads adjustment might disguise a large deformation in the transcriptome distribution, maps were adjusted for library size using the average scalar coefficient size factor determined by DESeq2. Resulting tracks were converted to bigWigs as ChIP‐seq tracks were, and the + and – tags from a given sample were plotted as overlays in a track hub.

### 2.13 ChIP‐seq

ChIP‐seq was performed as previously described.^8^ Briefly, ≈ 20 mg dorsal hippocampus from each mouse was minced on ice and cross‐linked with 1% formaldehyde for 10 minutes and quenched with 125 mM glycine for 5 minutes. Nuclei were prepared by Dounce homogenization of cross‐linked tissue in NIB (50 mM Tris‐HCl at pH 7.5, 25 mM KCl; 5 mM MgCl_2_, 0.25 M sucrose) with freshly added protease inhibitors and sodium butyrate. Nuclei were lysed in nuclei lysis buffer (10 mM Tris‐HCl at pH 8.0, 100 mM NaCl, 1 mM EDTA, 0.5 mM egtazic acid, 0.1% Na‐deoxycholate, 0.5% N‐lauroylsarcosine) with freshly added protease inhibitors and sodium butyrate and chromatin was sheared to ≈ 250 bp size using a Covaris S220 sonicator. Equal aliquots of sonicated chromatin were used per immunoprecipitation reaction with 4 µL H3K27ac antibody (Abcam, Cat. #4729; lot #GR323132‐1) preconjugated to Protein G Dynabeads (Life Technologies). Ten percent of the chromatin was saved as Input. ChIP reactions were incubated overnight at 4°C with rotation and washed three times in wash buffer. Immunoprecipitated DNA was eluted from the beads, reversed cross‐linked, and purified together with Input DNA. Ten nanograms of DNA (either ChIP or Input) were used to construct sequencing libraries using the NEBNext Ultra II DNA Library Prep Kit for Illumina (New England Biolabs, NEB). Libraries were multiplexed using NEBNext Multiplex Oligos for Illumina (dual index primers) and paired‐end sequenced (42 bp) on the NextSeq 550 platform (Illumina) in accordance with the manufacturer's protocol. ChIP‐seq tags generated with the NextSeq 550 platform were demultiplexed with the bcl2fastq utility and aligned to the mouse reference genome (assembly GRCm38/mm10) using Bowtie2 v2.3.4.1 with parameters –local ‐ X 1000). Aligned tags were sorted by tag ID using samtools v1.1 sort ‐n and polymerase chain reaction‐deduplicated using PICARD MarkDuplicates v2.21.3‐SNAPSHOT with parameters REMOVE_DUPLICATES = True ASSUME_SORT_ORDER = queryname. Peaks were detected using MACS2 (tag size = 42 bp; FDR < 1 × 10^−3^) from pooled H3K27ac tags of mice from the same condition along with treatment‐matched Input tags as control with the FDR controlled at 1% (‐q 0.01). The MTL method^36^ was used to compare H3K27ac enrichment in the four study conditions. Statistical significance of differential H3K27ac enrichments was assessed using RGT‐THOR v0.13.2 with default parameters. After BAM files were sorted by chromosome position using samtools sort and indexed using samtools index, two samples of WT PBS (K27‐362 and K27‐363) were compared to three samples of ACSS2 KO tau (K27‐321, K27‐322, K27‐323) with per‐replicate input adjustment. Differential enrichments reported by THOR were filtered for *P* values < 1 × 10^−2^⁰. UCSC Genome Browser track views were created for ChIP‐seq data by first pooling replicates and generating coverage maps using deepTools bamCompare v3.4.3 with parameter–operation subtract to adjust for the input signal and default parameters otherwise. Motif analysis of differential ChIP‐seq peaks was performed in the following way: to identify regulatory factors with an impact on gene expression, differential H3K27ac peaks gained with tau or lost with tau were first associated to the nearest transcriptional start site (TSS) using HOMER v4.6 annotatePeaks.pl. Peaks associated to the TSS of genes with significant expression differences in the double‐hit contrast (WT PBS vs. ACSS2 KO tau) were selected for further study. For peaks gained with tau that were filtered in this way, the peaks lost with tau were used as genomic background for motif detection, and vice versa. Motif detection was performed using HOMER findMotifsGenome.pl with parameters ‐size 200 and ‐mask. To identify *Rfx3* and *Rbpj1* target genes, motifs discovered through de novo detection were passed as input to HOMER findMotifsGenome.pl using parameters ‐find ‐size 300 ‐mask.

### 2.14 Single‐nuclei RNA‐seq and assay for transposase‐accessible chromatin using sequencing

Single‐nuclei dissociation was performed following the 10× Genomics user guide for nuclei isolation from complex tissues (CG000375 Rev B). Briefly, bilateral hippocampal tissue was homogenized in NP‐40 lysis buffer (10 mM Tris‐HCl, pH 7.4, 10 mM NaCl, 3 mM MgCl_2_, 0.1% NP‐40, 1 mM DTT) using biovortexer on ice. After 5 minutes of incubation, lysates were passed through a 70 µm Flowmi strainer and centrifuged at 500 × g for 5 minutes at 4°C. Pellets were washed and resuspended in ice‐cold PBS containing 1% bovine serum albumin (BSA). Nuclei were stained with ready‐made 7AAD solution (Sigma, Cat. SML1633) and sorted on MoFlo Astrios EQ (Beckman Coulter). Sorted nuclei were pelleted, resuspended in 0.1× lysis buffer (10 mM Tris‐HCl, pH 7.4, 10 mM NaCl, 3 mM MgCl_2_, 1% BSA, 1 mM DTT, 0.01% Tween‐20, 0.01% NP‐40, 0.001% digitonin) and incubated on ice for 2 minutes. Permeabilized nuclei were washed with wash buffer (10 mM Tris‐HCl, pH 7.4, 10 mM NaCl, 3 mM MgCl_2_, 1% BSA, 0.1% Tween‐20, 1 mM DTT) and resuspended in 1× diluted nuclei buffer with 1 mM DTT. All buffers contained 1 U/µL Sigma Protector RNase inhibitor (3335402001). Nuclei concentration was assessed via trypan blue staining and counting with a hemocytometer. This approach yielded ≈ 200,000 to 300,000 nuclei per brain. Library preparation was performed using Chromium Next GEM Single Cell Multiome ATAC + Gene Expression kit (10× Genomics) following the user guide (CG000338 Rev C). Targeted nuclei recovery rate was 10,000 nuclei/sample. For assay for transposase‐accessible chromatin using sequencing (ATAC‐seq), DNA and libraries were amplified for seven cycles using indexes from Sample Index Plate N, Set A. Library size distribution and concentration were measured using BioAnalyzer High Sensitivity DNA kit (Agilent) and NEB quantification kit. A total of 2.7 pM of pooled libraries were loaded onto 150‐cycle high‐yield Illumina flow cells and sequenced on Illumina NextSeq 550 using a custom recipe for read configuration 50:8:16:49. For RNA‐seq, cDNA was amplified for 9 cycles and libraries were amplified for 14 cycles using indexes from Dual Index Plate TT Set A. Library size distribution and concentration were measured using BioAnalyzer DNA1000 kit (Agilent) and NEB quantification kit. Pooled libraries 2.7 pM were loaded onto 150‐cycle high‐yield Illumina flow cells and sequenced on Illumina NextSeq 550 using read configuration 28:10:10:90.

### 2.15 Single‐nuclei RNA‐seq data processing and analysis

Multiomic datasets were processed independently. Single‐nuclei RNA‐seq (snRNA‐seq) data were generated using the 10× Cell Ranger^37^ pipeline (v6.0.2) with the mm10 genome. Specifically, fastq files were generated using cellranger mkfastq, followed by read quantifications using cellranger count with –include‐introns model suggested by 10× Genomics, and sample aggregation using cellranger aggr.

Downstream analysis was conducted in R (v4.3.2) and Seurat (v5.1.0)^38^, ^39^ with default parameters unless otherwise noted. Individual Seurat objects were generated for each replicate, processed using standard Seurat workflows including: SCTransform^40^, ^41^ for normalization, RunPCA for principal component analysis, and RunUMAP for uniform manifold approximation and projection. Doublet detection was performed with DoubletFinder (v3).^42^ Quality control filtering was applied to exclude cells with < 200 features or with mitochondrial read fractions > 5%. Then, the top 3000 most variable features were selected for replicate integration. Specifically, all Seurat objects from replicates were integrated using PrepSCTIntegration, followed by anchor identification with FindIntegrationAnchors, using reduction method reciprocal principal components analysis (rpca). The datasets were subsequently merged using IntegrateData.^43^ Downstream analyses included dimensionality reduction via RunPCA, batch effect correction with RunHarmony, and visualization using RunUMAP. Clustering was performed with FindNeighbors and FindClusters, and low‐quality clusters were removed. The final clustering resolution was set to 1. Phylogenetic trees were constructed using the BuildClusterTree function (Seurat). Cell percentage comparisons across cell types were assessed using the properller function from the speckle^44^ R package, which accounts for variability across biological replicates.

Differentially expressed genes (DEGs) were identified using FindAllMarkers for cluster‐wise comparisons and FindMarkers (Seurat) for pairwise comparisons, using a log2 fold‐change threshold of 0.25 and an adjusted *P* value cutoff of 0.05. DEG heatmaps were generated using DoHeatmap. Cell‐type annotations of the final used 24 clusters were assigned based on DEGs from cluster‐wise comparisons and further validated using Allen Institute Brain Atlas MAP MapMyCells.^45^ UMAP embeddings were visualized using DimPlot; violin plots for individual gene distributions were generated with VlnPlot. GO analysis of DEGs used Enrichr (https://maayanlab.cloud/enrichr‐kg).^46^ Other summary statistics were generated using R and ggplot.

Pseudopathology (pseudotime) trajectory analysis was performed using Monocle3^47^, ^48^ with default parameters. Two independent trajectory inferences were conducted, each using WT control centroids as root cells, defined separately along the *x* axis and *y* axis of the Uniform Manifold Approximation and Projection (UMAP) embedding. The trajectory was constructed using the following Monocle3 functions: preprocess_cds, reduce_dimension, cluster_cells, learn_graph, order_cells, and plot_cells. To identify key genes driving the trajectory, regression modeling was applied using fit_models, and differentially regulated genes were selected based on coefficient_table with adjusted *P* value threshold of 0.05.

Rfx (Rfx3/5/7) and Rpbj1 target genes were identified as above (see section 2.13). To expand the regulatory scope beyond Rfx3, motifs for other Rfx family members in were downloaded from the JASPAR database and added to the de novo motifs. To determine a suitable log‐odds similarity threshold for the database motifs, the consensus sequence was permuted to contain a number of mismatches based on the overall motif length, then those mismatching sequences were log‐odds scored against the position‐weight matrix and the first quartile value of the score distribution was chosen as the threshold. HOMER findMotifsGenome.pl was used again with command‐line parameter ‐find to scan both differential H3K27ac ChIP‐seq peaks at 1E‐10, 1E‐20, and single‐cell ATAC‐seq enriched regions or differential enriched regions to identify enhancers and other open loci with repressor binding moieties. Finally, the hits were annotated to nearby genes using HOMER annotatePeaks.pl and tables of likely target genes were produced. These target genes were overlapped with DEGs between WT PBS and KO tau across all clusters, and significance of overlap was determined via hypergeometric test.

Heatmaps were created by taking snATAC‐seq differential accessible regions (DARs) in all clusters at FDR < 0.1 (log‐ratio test) and combining them with bedtools merge. DARs were then scanned for the Rfx3 motif (result #2) Rbpj1 motif (result #5) using HOMER findMotifsGenome.pl with the ‐find parameter. Hits were annotated to the nearest gene using HOMER annotatePeaks.pl and measured for bulk log2(H3K27ac‐Input) signal using bigWigAverageOverBed. Finally, heatmaps were prepared by standardizing H3K27ac signals over the four study groups (ACSS2 WT‐PBS, WT tau, KO PBS, KO tau) and plotting with pheatmap, using the direction of gene expression from log2(KO tau/WT PBS) which had the greatest magnitude in any cluster in the single‐cell RNA‐seq as an annotation column.

### 2.16 Single‐nuclei ATAC‐seq data processing and analysis

Single‐nuclei ATAC‐seq (snATAC‐seq) data were processed using the 10× Cell Ranger ARC pipeline (v2.0.0) with the mm10 reference genome. Specifically, fastq files were generated using cellranger mkfastq, followed by read alignment and quantification using cellranger arc‐count.

Downstream analysis was conducted in R (v4.3.2), using Seurat (v5.1.0) and Signac (v1.14.0),^49^ with default parameters unless otherwise noted. Individual Seurat objects were generated for each replicate and processed using standard Signac workflows. Chromatin accessibility data were initialized using CreateChromatinAssay, incorporating peak calls from the Cell Ranger output. Genomic annotation was performed using GetGRangesFromEnsDb with EnsDb.Mmusculus. v79 from the UCSC database, followed by the creation of Seurat objects using CreateSeuratObject.

Seurat objects from all replicates were integrated using a standardized workflow. Integration anchors were identified with FindIntegrationAnchors using the reciprocal latent semantic indexing (rLSI) reduction method, followed by LSI embedding integration via IntegrateEmbeddings function. The union peak set was generated by merging peaks across replicates using the union function from the GenomicRanges^50^ package. This peak set was further refined by filtering with MACS2^51^ CallPeaks function based on the integrated object. Genomic annotation of peaks was performed using annotatePeak from the ChIPseeker^52^, ^53^ R package, with Gencode vM24 as the reference annotation database. Annotation distributions were visualized using plotAnnoBar from ChIPseeker.

Standard processing and normalization steps were performed for integrated objects using Signac as follows. Quality control metrics were computed and cells were filtered as follows: nCounts_peaks > 200, nFeature_peaks > 200 and < 30,000. Feature selection and normalization were performed using FindTopFeatures, RunTFIDF, and RunSVD, followed by clustering with FindClusters at a resolution of 1.0. Integration was conducted using FindIntegrationAnchors, IntegrateEmbeddings, and dimensionality reduction via RunUMAP. Phylogenetic relationships among clusters were inferred using BuildClusterTree.

To enable integration with snRNA‐seq data, gene activity scores for snATAC‐seq were computed on the co‐embedded Seurat object using GeneActivity, followed by assay creation with CreateAssayObject, normalization with NormalizeData, and scaling with ScaleData. The shared anchors between snATAC‐seq and snRNA‐seq datasets were identified using FindTransferAnchors, leveraging the most variable features from the snRNA‐seq dataset and using the canonical correlation analysis (CCA) method, followed by TransferData. The accuracy of predicted annotations for snATAC‐seq was assessed by examining unique cell counts per cell type and cluster. Heatmaps were generated using pheatmap^54^ to visualize annotation distributions. To co‐embed of snRNA‐seq and snATAC‐seq data, the datasets were merged using merge, followed by normalization, ScaleData, RunPCA, and RunUMAP. UMAP visualizations were generated using DimPlot, and summary statistics were computed in R and visualized using ggplot2.

DARs were identified using FindAllMarkers() for cluster‐wise comparisons and FindMarkers() for pairwise comparisons, using a likelihood ratio (LR) test. Regions were considered significantly differentially accessible with a minimum percentage threshold (min.pct) of 0.1, a log2 fold‐change threshold of 0.25, and a *P* value of < 0.001. Transcription factor (TF) motif enrichment analysis was performed using FindMotifs, and results were visualized with MotifPlot in Signac, using the JASPAR2020 database. Genome coverage tracks were generated using Signac visualization functions, including CovPlot, PeakPlot, TilePlot, and AnnotationPlot.

## 3 RESULTS

### 3.1 ACSS2 KO exacerbates learning and memory impairments induced by AD tau

Injection of human pathological AD tau (tau) isolated from *post mortem* brains of AD patients induces AD‐like pathology in mice, characterized by hyperphosphorylation of endogenous tau and spreading of the pathology.^29^, ^55^ Given the central role of tau in AD and other tauopathies, we used this model to determine whether associated molecular and behavioral changes are worsened by loss of ACSS2.

**FIGURE 1:**
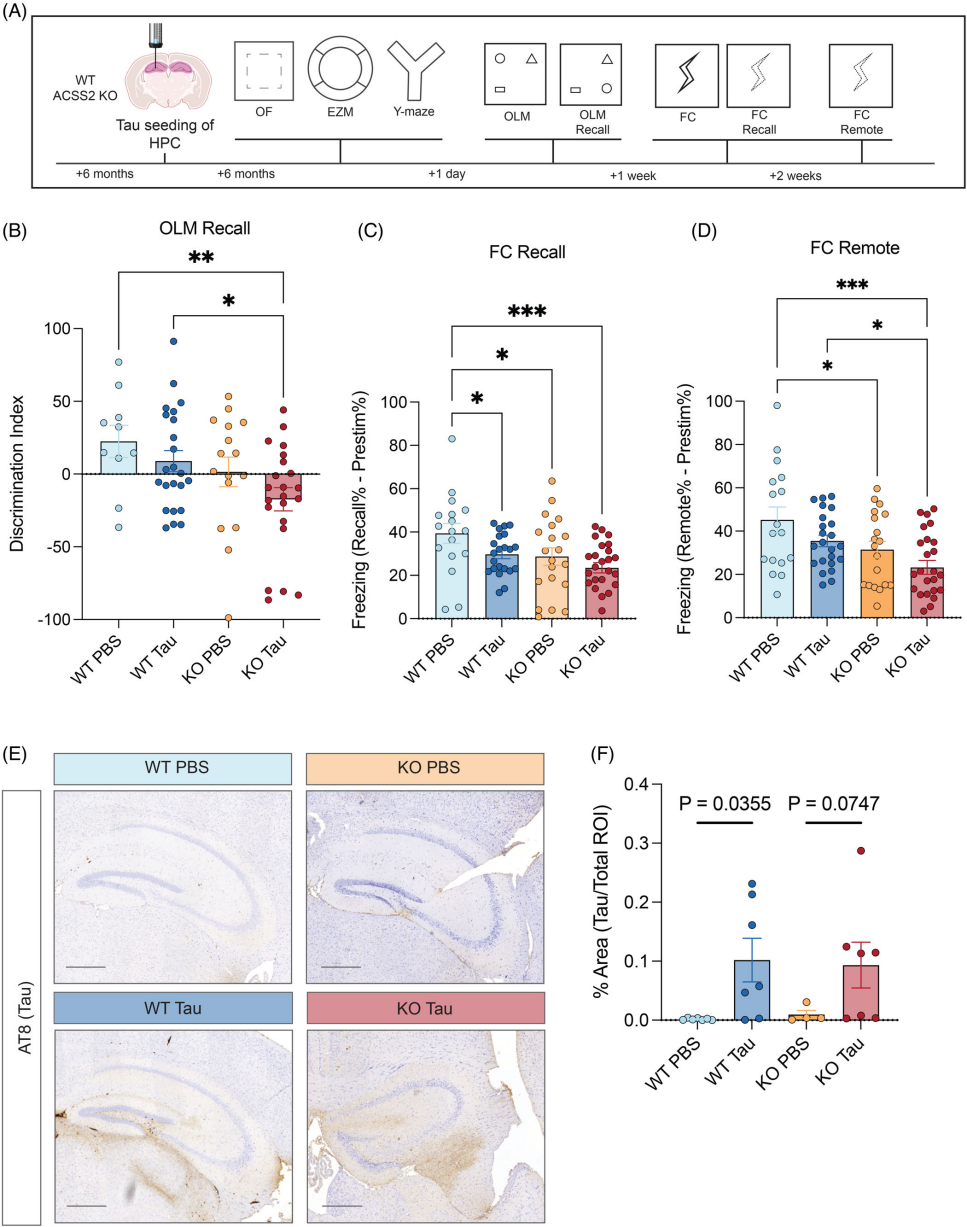
ACSS2 deletion exacerbates learning and memory impairments induced by hippocampal AD tau injection. A, Schematic of behavioral battery performed on AD‐Tau injected WT and ACSS2 KO mice along with PBS‐injected controls. Battery includes: tests of baseline behavior (open field [OF], and elevated zero maze [EZM]), tests of working memory (Y‐maze), tests of long‐term spatial memory (object location memory [OLM]), and long‐term fear memory (contextual fear conditioning [FC]). B, Decreased discrimination index of moved objects in AD tau–injected ACSS2 KO mice indicate more severe impairments of learning compared to other groups. One‐way ANOVA (*F*_3,67_ = 3.281, *P* = 0.0261) with Fisher LSD post hoc (*P < 0.05, **P < 0.01, ***P < 0.001). Non‐significant comparisons not shown. Bar graphs represent mean ± standard error of the mean. Points represent individual mice (WT PBS *n* = 10; WT tau *n* = 23; KO PBS *n* = 16; KO tau *n* = 22). C, Freezing levels at 24 hour recall show severe memory impairments in AD tau–injected and ACSS2 KO mice, with largest amplitude of decrease in the double‐hit KO tau mice. One‐way ANOVA (*F*_3,80_ = 4.001, *P* = 0.0104) with Fisher LSD post hoc (*P < 0.05, **P < 0.01, ***P < 0.001; WT PBS *n* = 17; WT tau *n* = 22; KO PBS *n* = 20; KO tau *n* = 24). D, Freezing levels at 2 weeks remote recall show more severe memory impairments in AD tau–injected ACSS2 KO mice compared to other groups. One‐way ANOVA (*F*_3,80_ = 5.592, *P* = 0.0016) with Fisher LSD post hoc (*P < 0.05, **P < 0.01, ***P < 0.001). E, AT8 immunohistochemistry showing similar levels of endogenous tau phosphorylation in AD tau–injected WT and ACSS2 KO hippocampi. F, Quantification of area occupied by AT8 staining within the DG of AD tau–injected WT and ACSS2 KO hippocampi. Brown–Forsythe ANOVA (F* = 3.668 [3.0, 12.25], *P* = 0.0431) with post hoc unpaired *t* with Welch correction (*P < 0.05, **P < 0.01, ***P < 0.001; WT PBS *n* = 7; WT tau *n* = 7; KO PBS *n* = 4; KO tau *n* = 7). ACSS2, acetyl‐CoA synthetase 2; AD, Alzheimer's disease; ANOVA, analysis of variance; DG, dentate gyrus; KO, knockout; LSD, least significant difference; PBS, phosphate‐buffered saline; ROI, region of interest; WT, wild type.

To examine the role of ACSS2 in tauopathy‐associated cognitive dysfunction, we performed AD tau injections into the hippocampus of WT and ACSS2 KO mice and performed a battery of behavioral tests 6 months post‐injection, to characterize learning and memory impairments associated with hippocampal tau injection (Figure 1A). As reported previously for tau injection^55^ and for ACSS2 KO,^22^ we found no evidence of alterations in anxiety‐like behavior via open field (Figure S1A in supporting information) or elevated zero maze assays (Figure 1B), and no impairment of short‐term memory in Y‐maze (Figure 1C). Further, locomotor activity was not affected in any of the behavioral assays (Figure 1A–C). These results emphasize the lack of gross behavioral abnormalities in the ACSS2 KO mice injected with AD tau.

We then examined long‐term memory using recall tests of spatial memory. Object location memory (OLM) measures recall of long‐term spatial memories 24 hours after conditioning, requiring the mouse to remember an initial arrangement of items to identify a displaced object (Figure 1A). Fear conditioning (FC) measures long‐term associative memories forming between an aversive stimulus (foot shock) and spatial cues from a novel experimental chamber (Figure 1A). We previously found that ACSS2 KO mice^22^ and mice with dorsal hippocampal knockdown of ACSS2^21^ display impaired performance in these tasks.

Strikingly, ACSS2 KO mice combined with AD tau injection showed impairments of long‐term memory in both OLM (Figure 1B) and contextual FC (Figure 1C, D). With OLM, the PBS‐injected WT mice (WT PBS) favored exploration of the object that had been moved on the test day, demonstrating intact long‐term spatial memory. However, the discrimination index was decreased by tau seeding and ACSS2 loss (one‐way analysis of variance [ANOVA] *F*_3,67_ = 3.281, *P* = 0.0261). Post hoc comparisons revealed that this effect was primarily driven by the double hit of AD tau injection in ACSS2 KO background (KO tau). AD tau–injected WT (WT tau; Fisher LSD *P* = 0.3363) or PBS‐injected ACSS2 KO mice (KO PBS; Fisher LSD *P* = 0.1639) showed a non‐significant trend for decreased discrimination index (Figure 1B). Memory was more severely impacted in KO tau mice, with a significant decrease of discrimination index compared to both WT PBS (Fisher LSD *P* = 0.0062) and WT tau mice (Fisher LSD *P* = 0.0201; Figure 1B, right).

This pattern of long‐term memory deficits was mirrored in mice that underwent FC. Both tau injection and ACSS2 KO resulted in diminished recall of contextual cues 24 hours after acquisition (one‐way ANOVA, *F*_3,80_ = 4.001, *P* = 0.0104; Figure 1C). However, post hoc multiple comparisons indicated that group differences were mostly driven by significant decrease of freezing in the double hit KO tau mice compared to PBS‐injected WT mice (Fisher LSD, *P* = 0.0049), although both tau injection (Fisher LSD, *P* = 0.0441) and ACSS2 KO alone (Fisher LSD, *P* = 0.0295) showed reduction compared to WT PBS controls (Figure 1C). Additionally, this deficit persisted into remote recall (one‐way ANOVA, *F*_3,80_ = 5.592, *P* = 0.0016, Figure 1D), with the KO tau mice continuing to show decreased freezing 14 days after acquisition (Fisher LSD, *P* = 0.0001). Similarly to OLM, KO tau mice showed a significant memory impairment beyond what was observed in WT tau mice (Fisher LSD, *P* = 0.0177).

To test whether the observed behavioral impairments are accompanied by more severe neuropathology, we injected AD tau into the hippocampus of ACSS2 KO mice^22^ and WT controls, and assessed phospho‐tau levels by immunofluorescence 6 months post‐injection, because pathological tau becomes hyperphosphorylated during aggregation and spreading.^29^ As expected, tau phosphorylation occurred in the dentate gyrus (DG) of tau‐injected mice, but not in PBS‐injected littermates (Figures 1E, F). Notably, in the ACSS2 KO mouse, AD tau seeding induced hyperphosphorylation of endogenous tau to a similar extent as in WT mice (Figures 1E, F), indicating that there was no worsening of tau spreading or accumulation with ACSS2 loss.

Together, these findings suggest that, while individual AD tau injection and ACSS2 KO both impair long‐term memory formation, these deficits are exacerbated with the “double hit” of AD tau seeding in animals deficient in ACSS2. Across all assays of long‐term memory we used, KO tau mice consistently exhibited the poorest memory performance among all groups. Because our evidence shows that short‐term memory, anxiety‐like behavior, and general locomotion were not affected, these results indicate that ACSS2 loss selectively contributes to more severe impairment of long‐term memory in this model of tauopathy. The worsening of behavioral phenotypes in KO tau mice was independent of phospho‐tau levels in the dorsal hippocampus, indicating contributions from other molecular pathways.

### 3.2 Epigenetic and transcriptional profiling of AD tau–injected ACSS2 KO mice identify aberrant de‐repression of Rfx3 target genes

Given the recently uncovered role of ACSS2 in gene regulation in the brain,^21^, ^22^ we next examined gene expression and histone acetylation implicated in human AD.^8^, ^9^ Mouse brain tissue exhibits peak tau pathology at 6 months post‐injection;^29^ thus, we focused on this time point.

**FIGURE 2:**
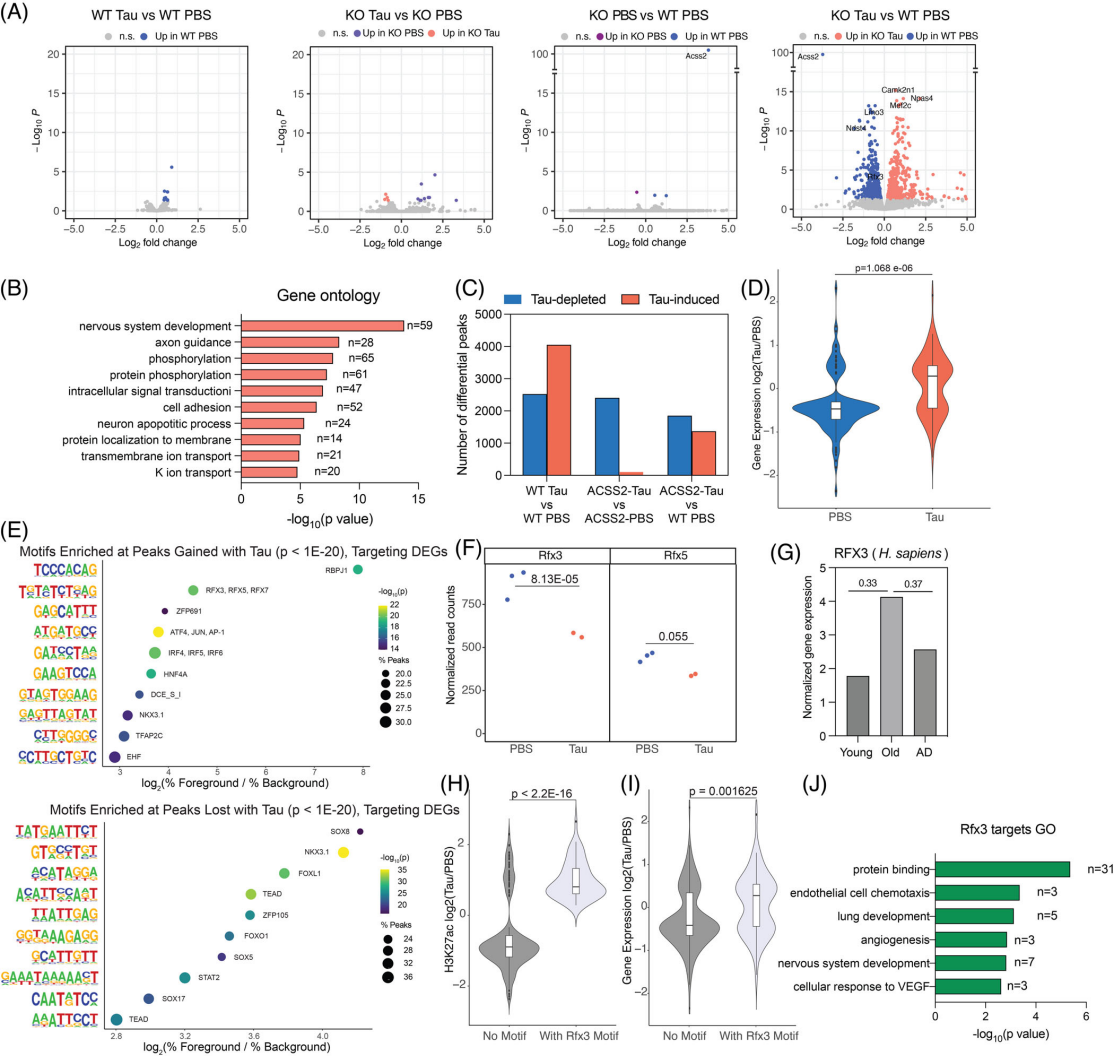
Epigenetic and transcriptional profiling of the AD tau–injected ACSS2 KO hippocampus. A, Bulk RNA‐seq showing severe transcriptional dysregulation in the hippocampus of AD tau–injected ACSS2 KO mice (DESeq2 with Wald test, *P*adj < 0.05; WT PBS *n* = 3; WT tau *n* = 3; KO PBS *n* = 3; KO tau *n* = 2). B, GO analysis of DEGs in AD tau–injected ACSS2 KO mice compared to PBS‐injected WT controls. C, Number of differential H3K27ac peaks in different comparisons. D, Expression of genes near H3K27ac peaks induced or depleted by AD tau injection. E, Motif analysis reveals the presence of Rfx3 binding sites at AD tau–induced peaks in ACSS2 KO hippocampus. F, *Rfx3* mRNA expression is significantly decreased in the hippocampus of AD tau–injected ACSS2 knock‐out mice. G, H3K27ac enrichment is significantly increased at Rfx3 binding sites in AD tau–injected ACSS2 KO mice compared to binding sites without the Rfx3 motif. H, The expression of genes near Rfx3 binding sites is significantly increased by AD tau injection in ACSS2 KO mice. I, GO analysis of Rfx3 target genes induced by AD tau in ACSS2 KO hippocampus. ACSS2, acetyl‐CoA synthetase 2; AD, Alzheimer's disease; DEG, differentially expressed gene; GO, Gene Ontology; KO, knockout; PBS, phosphate‐buffered saline; WT, wild type.

Intriguingly, bulk RNA‐seq of hippocampal tissue revealed that the double‐hit combination of AD tau injection and loss of ACSS2 led to a striking increase of transcriptional dysregulation (Figure 2A, right). Thus, while there were limited DEGs either in AD tau injection in WT mice or in the ACSS2 KO mice, comparison of WT PBS–injected mice to double hit ACSS2 KO tau–injected littermates resulted in 1029 DEGs (WT PBS = 533; KO tau = 496; Figure 2A, right) with a similarly large difference between tau‐injected WT and KO mice (1378 DEGs, WT tau = 744; KO tau = 634; Figure S2A in supporting information), exhibiting an overlapping set of genes (Figure 2B). These results (Figure 2A, left and middle panels) confirmed our previous findings that ACSS2 KO alters only a few genes at baseline in the absence of a memory task.^22^ GO analysis showed that double hit KO tau dysregulated genes were related to nervous system development, axon guidance, neuronal apoptosis, and ion channels (Figure 2B), suggesting broad impairment of neuronal function. In line with findings linking ACSS2 to the regulation of immediate early genes,^7^, ^8^ this family of genes was strongly represented among the DEGs, with *Npas4* being the most significantly dysregulated transcript by the double hit other than Acss2 itself (Figure 2A, right).

We then performed ChIP‐seq to profile the genome‐wide enrichment of H3K27ac, a histone acetylation mark strongly dysregulated in human AD. We first examined the contrast between WT PBS and WT tau conditions and noted substantial dysregulation of H3K27ac peaks, with moderate gains in the tau‐injection model resembling the observed increase in human *post mortem* AD patient brains^9^ (1378 PBS‐specific, 2194 tau‐specific; Figure S2B, C). H3K27ac gains and losses were associated with known neurodegeneration‐related genes, including at the Hnrnpr^56^ and Deptor loci^57^ (Figure S2D). GO analysis of genes associated with tau‐specific H3K27ac peaks revealed enrichment of genes related to the regulation of axonogenesis, neurological system processes, long‐term synaptic depression, and the regulation of axonal function and synaptic plasticity (Figure S2E); these processes may contribute to the tau‐related learning and memory impairments in this model (see Figure 1) in spite of modest changes in DEGs.

We then performed H3K27ac ChIP‐seq in WT and ACSS2 KO mice with AD tau injection. As before, there were thousands of differential peaks and tau injection in WT mice increased histone acetylation (Figure 2C, left), which may reflect similar chromatin changes at disease‐driving genes as in human *post mortem* brain.^8^, ^9^ In stark contrast, loss of H3K27ac peaks predominated in the ACSS2 KO background (Figure 2C, middle), and in the double hit comparison (Figure 2C, right), indicating differential impact of AD tau injection on the hippocampal epigenome with loss of ACSS2. Given the exacerbated behavioral (Figure 1) and transcriptional (Figure 2A, right) impairments in KO tau relative to the WT PBS mice, we further analyzed the double hit comparison. As expected, there was significant association of H3K27ac peaks with gene expression, with gene expression decreases linked to H3K27ac peak losses, and gene expression increases linked to H3K27ac peak gains (Figure 2D). We investigated transcription factors that may be driving the gene expression changes in response to AD tau within the ACSS2 KO mice by analyzing *de novo* DNA motifs under the differential H3K27ac peaks that likely regulate those genes (each differential peak was associated to the nearest TSS, and peaks associated to double hit DEGs were selected for motif analysis). Peaks lost in KO tau mice were enriched for Sox8 (Figure 2E, bottom), which plays a critical role in remyelination after toxin‐mediated demyelination in mice.^58^ Peaks gained in the double‐hit KO tau mice were enriched for motifs of Rbpj1, a TF with dual repressor/activator activity related to Notch signaling,^59^ as well as for motifs of the Rfx family of transcriptional repressors; these motifs were present in 26% and 25% of all KO tau–enriched peaks, respectively (Figure 2E, top). The *Rfx3* gene has frameshift mutations linked to neurodevelopmental disorders,^60^ and the expression of *Rfx3* was significantly decreased in the hippocampus of the double‐hit KO tau mice, with *Rfx5* also showing decreased expression (Figure 2F). Strikingly, both H3K27ac enrichment (Figure 2G) and gene expression (Figure 2H) of *Rfx3* gene targets were significantly increased in the double‐hit KO tau mice, as predicted given that the Rfx family members are transcriptional repressors and their expression was decreasing in KO tau mice. Notably, neither Rfx3 nor Rbpj1 target genes are typically related to neuronal function, and consistently, the top enriched GO categories included protein binding, endothelial cell chemotaxis, and lung development (Figure 2I). Thus, aberrant de‐repression of Rfx3 target genes in AD tau–injected ACSS2 KO mice might contribute to impairment of neuronal and cognitive functions observed in this model, though more rigorous testing is needed (see Figure 1).

Overall, these results indicate that the double‐hit KO tau mice exhibit dysregulation of histone acetylation and gene expression in the dorsal hippocampus. This is in line with the impairments of long‐term memory and point to a potential key role of transcriptional and epigenetic mechanisms in disease etiology.

### 3.3 Cell type–specific profiling of gene expression and chromatin accessibility in the hippocampus of AD tau–injected ACSS2 KO mice

**FIGURE 3:**
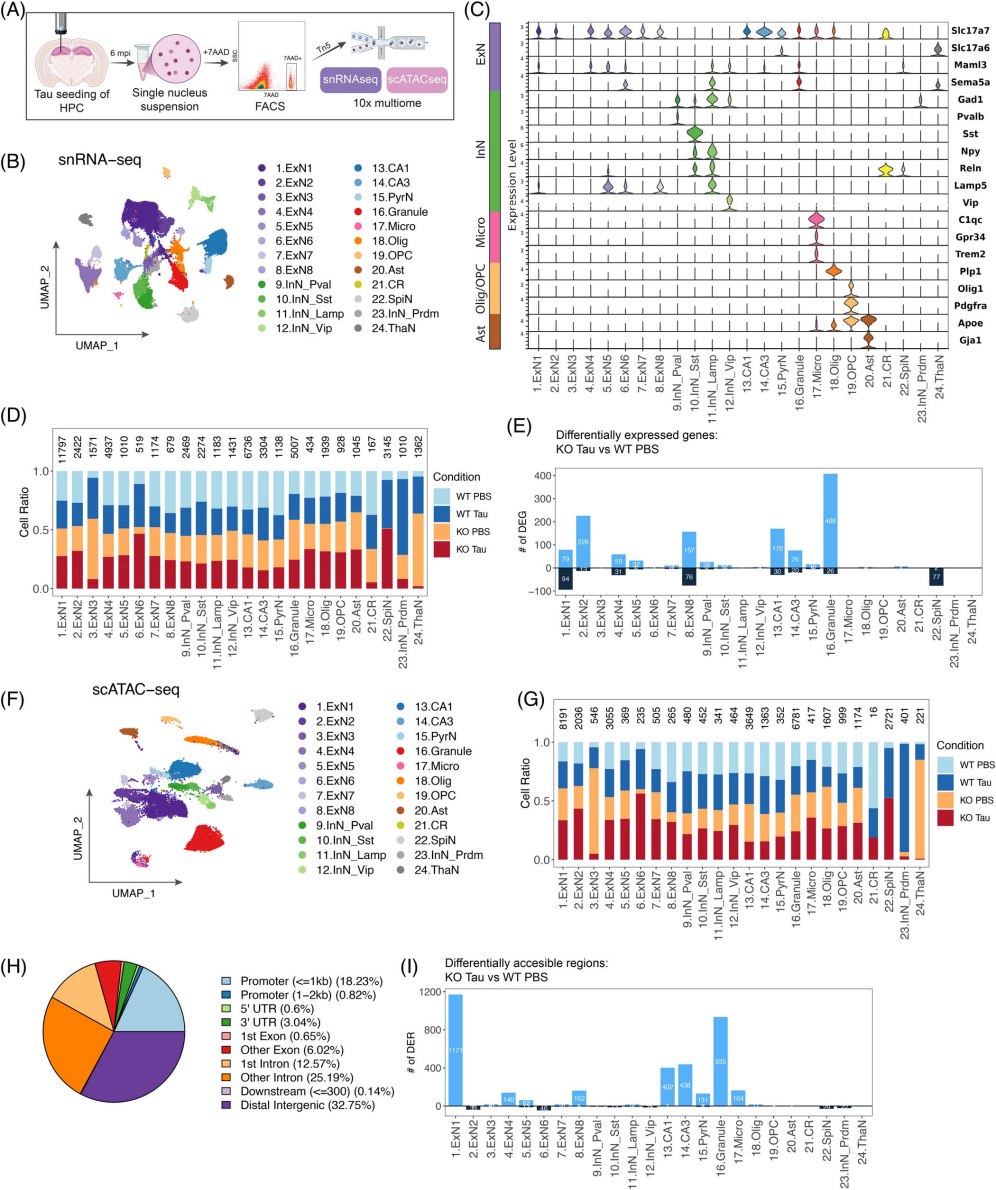
Single‐nuclei profiling of gene expression and chromatin accessibility of AD tau–injected ACSS2 KO mice. A, Schematic detailing single‐nuclei sequencing workflow. B, UMAP embedding of neuronal and non‐neuronal cell types identified in snRNA‐seq of mouse hippocampus (*n* = 3 replicates per condition; two pooled mice per replicate). C, Violin plot showing expression of known marker genes (curated from the literature) across cell populations. D, Changes in cell number across conditions (genotype, injection) in the snRNA‐seq dataset. Clusters 22 to 24 likely constitute dissection artefacts. E, Differentially expressed genes across all clusters between WT PBS and KO tau. F, UMAP embedding of neuronal and non‐neuronal cell types identified in snATAC‐seq of mouse hippocampus. G, Changes in cell number across conditions (genotype, injection) in snATAC‐seq dataset. Number of cells in each cluster indicated by number above barplot, and all columns presented as percentage of whole. H, Pie chart showing genomic location of snATAC‐seq peaks across all conditions. I, Differentially accessible regions across all clusters in AD tau–injected ACSS2 KO mice compared to PBS‐injected WT controls. ACSS2, acetyl‐CoA synthetase 2; AD, Alzheimer's disease; KO, knockout; PBS, phosphate‐buffered saline; snATAC‐seq, single‐nuclei assay for transposase‐accessible chromatin using sequencing; snRNA‐seq; single‐nuclei RNA sequencing; UMAP, Uniform Manifold Approximation and Projection; WT, wild type.

These findings highlight important behavioral, transcriptional, and epigenetic changes in the hippocampi of ACSS2 KO mice in response to AD tau injection, along with potential aberrant TF activity. To determine whether there were specific cell populations most affected by AD tau and ACSS2 KO, we used the 10x Genomics MultiOmics platform to assess transcription (RNA‐seq) and chromatin accessibility (ATAC‐seq) profiles from single nuclei (Figure 3A). A total of 57,681 nuclei were sequenced from the hippocampi of PBS‐ or tau‐injected ACSS2 KO mice and WT controls (*n* = 3 replicates per condition, with each replicate comprising hippocampi pooled from two mice). Initial clustering yielded 36 subgroups (Figure S3A, B in supporting information), which were mapped onto annotated cell types using MapMyCells (Allen Brain Atlas;^45^ Table S1 in supporting information). Similar cell types were merged to yield 24 clusters corresponding to major neuronal and non‐neuronal cell types in the hippocampus (Figure 3B), and cluster identities were confirmed via published cell‐type markers (Figure 3C), with analysis of top cluster‐specific DEGs (Figure S3C).

The majority of cell types were relatively stable across the four conditions (Figure 3D, Figure S3D); exceptions included clusters 22 to 24, comprising medium spiny neurons (MSN), Prdm12 inhibitory neurons (Prdm12 InN), and thalamic neurons (Thal). These populations were likely dissection artifacts, as these cell types are not generally found in the hippocampus,^45^ and exhibited a strong bias toward the second replicate across all three populations (Figure S3E, F). Thus, we removed these clusters from subsequent analysis.

Transcriptionally, cell types showed varying levels of RNA alterations across the possible comparisons (Figure 3E, Figure S3G). Consistent with the bulk RNA‐seq dataset (Figure 2A), the contrast between PBS‐injected WT and tau‐injected ACSS2 KO mice showed the most divergence, with seven clusters exhibiting < 50 DEGs (*P*adj < 0.1; Log2FC > 0.25; percent expression within cluster > 10%; Figure 3E), compared to ≤ 6 for the remaining contrasts of tau alone or ACSS2 KO alone (Figure S3G). Overall, a greater number of DEGs were identified across all contrasts than by bulk RNA‐seq, emphasizing cell type–specific effects and possibly pointing to a difference between the mature, polyadenylated transcripts selected by bulk analyses, and the nuclear transcripts identified by snRNA‐seq, though shared DEGs between bulk and pseudobulked snRNA‐seq were positively correlated (*R* = 0.57, *P* < 0.001; Figure S3H). The most heavily affected clusters across groups were all excitatory neurons, specifically excitatory neurons 1, 2, 4, and 8, as well as CA1 and CA3 pyramidal cells (PCs) and the granule cells (GCs) of the DGs (Figure 3E). Notably, Acss2 transcript levels were not enriched in any specific cluster (Figure S3I).

In addition to RNA, we examined DNA from 47,672 of the nuclei to measure chromatin accessibility, and labeled populations based the previously mapped snRNA‐seq dataset above (Figure 3F, Figure S4A, B in supporting information), confirming cluster identity using MapMyCells.^45^ Overall, the UMAP projection of the joint gene expression/chromatin accessibility profiles recapitulated clustering based on either modality alone (Figure S4C), and similar ratios of cells were recovered between approaches (Figure 3D, G and Figure S4D, E), although the WT PBS condition was slightly undersampled in the snATAC‐seq (Figure S4F). The largest singular percentage (33%) of called peaks mapped to distal intergenic regions with 18% mapping within 1 kb of promoters, and a total of 44% to other regions in the gene body (7% in exons and 38% in introns; Figure 3H). As in the snRNA‐seq data, the majority of DARs between WT PBS and KO tau (*P*val < 0.001, Log2FC > 0.25; percent expression within cluster > 10%) mapped to GCs and PCs, with significant contributions from excitatory neuronal clusters 1, 4, and 8 (Figure 3I). Contrasts involving the KO tau condition exhibited substantially more DARs than contrasts without KO tau (Figure S4D), suggesting that, surprisingly, loss of ACSS2 leads to increased chromatin openness in the presence of tau. As ACSS2 is directly linked to increased histone acetylation, we posit that this effect is potentially driven by downstream indirect pathways affected in this long‐term, steady‐state model.

Together, our multiomic single‐nuclei profiling approach robustly captured major neuronal and non‐neuronal cell populations of the hippocampus, and provided converging evidence for a cell type–specific dysregulation of gene expression and chromatin accessibility in the tau‐seeding model. Similar to the bulk RNA‐seq and behavioral outcomes, the double‐hit KO tau mice were more severely impacted compared to single hit WT tau and KO PBS animals.

### 3.4 AD tau–injected ACSS2 KO mice exhibit transcriptional dysregulation along the hippocampal trisynaptic circuit and perforant path

**FIGURE 4:**
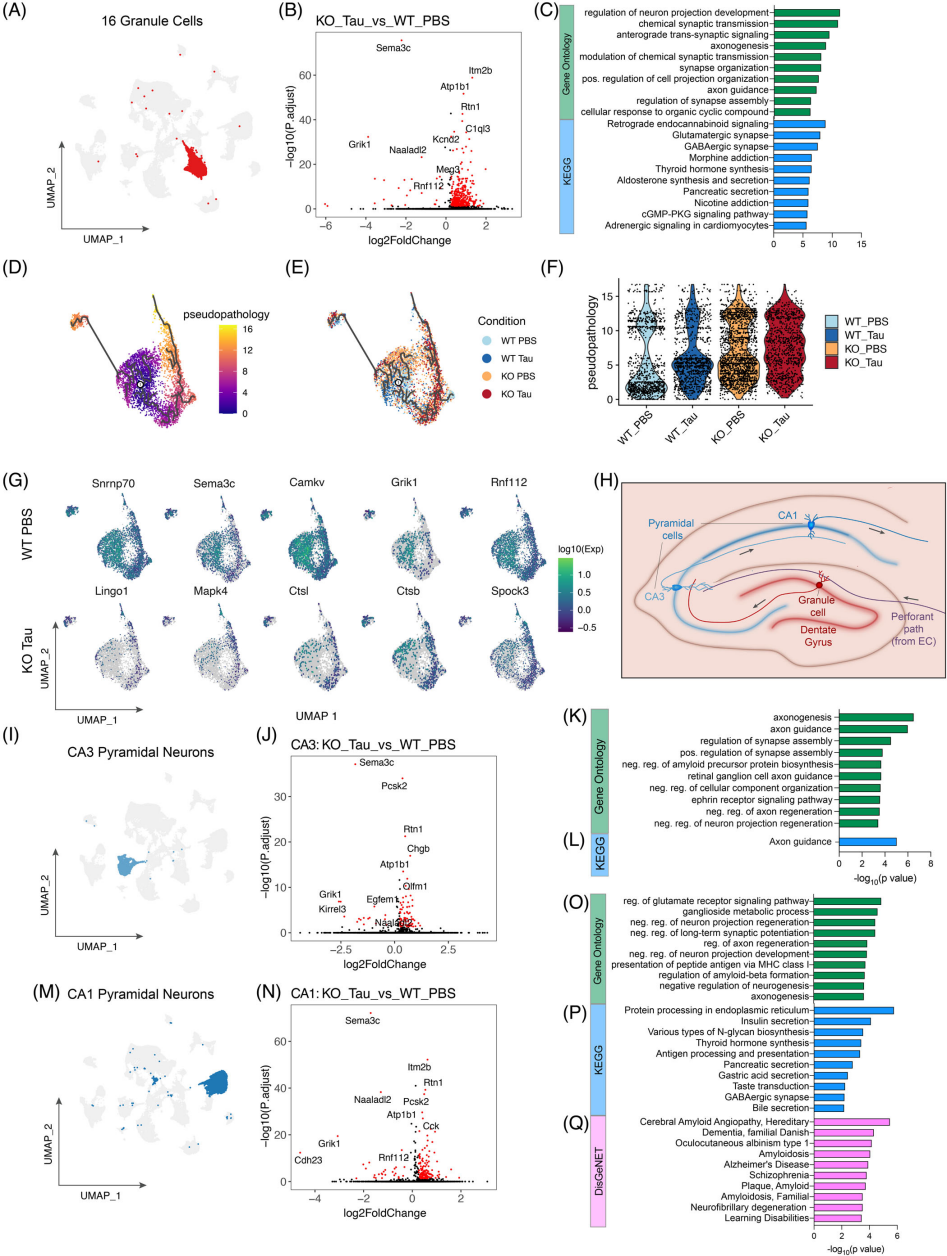
AD tau–injected ACSS2 KO mice exhibit transcriptional dysregulation of hippocampal circuitry. A, Location of GCs on the UMAP projection. B, Volcano plot showing DEGs between PBS‐injected WT mice (WT Ctrl) and tau‐injected ACSS2 KO mice (KO tau; *P*adj < 0.1). C, Top, Top 10 GO terms (*q* value < 0.1) for DEGs (*P*adj < 0.1) upregulated in KO tau. Bottom, Top 10 KEGG pathways (*q* value < 0.1) for DEGs (*P*adj < 0.1) upregulated in KO tau. D, UMAP projection of the GC cluster colored by pseudopathology score (Monocle3). Starting node (centroid of WT PBS condition) labeled (1). Gray lines delineate the pseudopathology trajectory. E, UMAP projection of the GC cluster colored by condition. Starting node (centroid of WT PBS condition) labeled (1). Gray lines delineate the pseudopathology trajectory. F, Violin plot of individual cells plotted by pseuopathology score. G, Individual genes defining the pseudopathology trajectory mapped by expression across individual cells in the UMAP projection. Top, Genes defining early pathology (higher expression close to the start node). Bottom, Genes defining later pathology (higher expression near termini). H, Diagram of key neuronal populations within the perforant path, and their anatomical location in the dorsal hippocamps. I, Location of pyramidal cells within the CA3 subfield of the hippocampus (CA3) on the UMAP projection. J, Volcano plot showing DEGs between PBS‐injected WT mice (WT Ctrl) and tau‐injected ACSS2 KO mice (KO tau; *P*adj < 0.1). K, Top 10 GO terms (*q* value < 0.1) for DEGs (*P*adj < 0.1) upregulated in KO tau. L, Significant KEGG pathways (*q* value < 0.1) for DEGs (*P*adj < 0.1) upregulated in KO tau. M, Location of pyramidal cells within the CA1 subfield of the hippocampus (CA1) on the UMAP projection. N, Volcano plot showing DEGs between PBS‐injected WT mice (WT Ctrl) and tau‐injected ACSS2ko mice (KO tau; *P*adj < 0.1). O, Top 10 GO terms (*q* value < 0.1) for DEGs (*P*adj < 0.1) upregulated in KO tau. P, Significant KEGG pathways (*q* value < 0.1) for DEGs (*P*adj < 0.1) upregulated in KO tau. Q, Top 10 disease terms (*q* value < 0.1) for DEGs (*P*adj < 0.1) upregulated in KO tau (DisgeNET). ACSS2, acetyl‐CoA synthetase 2; AD, Alzheimer's disease; DEG, differentially expressed gene; GC, granule cell; GO, Gene Ontology; KEGG, Kyoto Encyclopedia of Genes and Genomes; KO, knockout; PBS, phosphate‐buffered saline; UMAP, Uniform Manifold Approximation and Projection; WT, wild type.

We then investigated transcriptional changes within cell clusters to uncover deeper effects on pathways. The cluster with the largest number of DEGs between PBS‐injected WT (WT Ctrl) and tau‐injected ACSS2 KO (KO tau) mapped to GCs of the DGs (434 DEGs, *P*adj < 0.1; Log2FC > 0.25; percent expression within cluster > 10%; Figure 3E, Figure 4A, B). The DG also featured the highest degree of phospho‐tau staining, particularly in the hilus and molecular layer (Figure 1E), which play key roles in hippocampal information processing and associative memory formation. Of the 26 genes downregulated in the KO tau condition, the most robustly perturbed included semaphorin 3c (*Sema3c*), a positive regulator of axon guidance, and the glutamate ionotropic receptor kainate‐1 (*Grik1*; Figure 4B). Far more genes (408) were upregulated toward the KO tau condition and these, too, were enriched in gene ontologies and pathways involved in neuronal projections and synaptic function (Figure 4C). This unexpected effect of ACSS2 loss leading to increased gene expression was in line with the increased chromatin accessibility, and was potentially linked to decreased expression of repressors (e.g., RFX3 and Rbpj1; see Figure 2E–J) and other mechanisms downstream of ACSS2.

To examine how tau injection and ACSS2 KO impacted transcriptional profiles on a continuous pathological trajectory, we extracted the GC cluster and performed pseudotime (or, in this case, pseudopathology) analysis using Monocle3.^47^, ^61^ We set the root node as the cell closest to the centroid of the WT PBS distribution, assuming this point best represented the least pathological transcriptional state. Compared to the root node, the KO tau cell cluster predominantly shifted right along UMAP 1 (Figure 4E, Figure S5A in supporting information) and this shift was evident on the pseudopathology trajectory, with more KO tau cells at the extremities of the trajectory, and WT tau and KO PBS occupying intermediate states (Figure 4E, F). As in the pairwise comparison, genes enriched early in the pseudopathology trajectory were associated with axonogenesis (*Snrnp70*,^62^ *Sema3e* ^63^, ^64^), as well as enhanced synaptic function (*Camkv*,^65^ *Grik1* ^66^). We also noted early enrichment of RING finger protein 112 (Rnf112), which negatively regulates neuronal inflammation,^67^ and promotes synaptic plasticity.^68^ In contrast, genes that increased along the pseudopathology trajectory and thus in the KO tau GCs included: *Lingo1*, a negative regulator of neurite outgrowth and myelination,^69^ and a putative therapeutic target in AD;^70^ *Mapk4*,^71^ cathepsins (*Ctsl* and *Ctsb*)^72^, and *Spock3* ^73^ (Figure 4G).

In contrast to the relatively balanced chromatin landscape observed in the full snATAC‐seq dataset (Figure 3H), the majority of DARs (93.8%) between WT PBS and KO tau mapped to gene promoters (Figure S5B). Changes in accessibility at these sites was mild, with DARs^74^ primarily occurring at already open promoters, including those of Serinc1, an activity‐dependent lipid carrier,^74^ and Trim44, an enhancer of autophagy^75^ (Figure S5C). This chromatin profile suggests that the impact of KO tau on GCs is driven by the exacerbation of existing transcriptional patterns rather than the gain of disease‐specific promoters and enhancers.

The GCs of the DGs receive inputs from the entorhinal cortex, forming the first hippocampal node of the perforant path.^76^ GCs project onto the PCs of the CA3, which in turn project onto the CA1, forming the trisynaptic circuit (Figure 4H). Given the number of synaptic genes dysregulated in the GCs, we examined the transcriptional profiles of the downstream CA3 and CA1 PCs. Similarly to GCs, CA3 cells (Figure 4I) showed downregulation of *Sema3c* and *Grik1* in the KO tau condition (Figure 4J), and many of the top GO terms^77^ and Kyoto Encyclopedia of Genes and Genomes (KEGG) pathways reflected axonogenic and synaptic dysregulation (Figure 4K, L). However, we also noted terms indicative of increased pathology, including negative regulation of amyloid precursor protein (APP) production, and negative regulation of axon regeneration (Figure 4K). Interestingly, the top GO term for genes associated with the non‐diseased state (WT PBS) was activation of protein kinase B, a major neuroprotective pathway, which exerts antiapoptotic effects^78^, ^79^ and axonal regeneration in vitro,^80^ and in models of Parkinson's disease^81^ (Figure S5D). In contrast to GCs, the CA3 PCs show increased DARs at intronic (12.1% and 25.3%) and distal intergenic regions (23.29%), though the small majority remain promoter proximal (27.63%; Figure S5E).

KO tau CA1 PCs (Figure 4M), once again downregulated *Sema3c* and *Grik1* (Figure 4N), but their top 10 GO terms showed increased representation of disease‐related processes compared to the CA3, including negative regulation of neuronal projection regeneration, long‐term potentiation, and neurogenesis, as well as Aβ formation (Figure 4O). The top KEGG pathway (protein processing in the endoplasmic reticulum), also indicated increased stress on the CA1 (Figure 4P). GO terms biased toward the WT PBS state featured terms associated with intact neuronal function (equilibrioception, sensory perception), and mitochondrial function (aerobic electron transport chain; Figure S5F). DAR annotation in these cells was again primarily promoter proximal (Figure S5G). Finally, we examined the disease associations of the upregulated genes,^82^ and found almost exclusive representation of neurodegenerative diseases, including AD and other forms of familial amyloidoses (Figure 4Q), terms that were not significantly enriched (*q* value < 0.1) in KO tau‐biased genes of the GC and CA3 (Figure S5H; no significant DisgeNET terms were uncovered for KO tau‐biased genes in the CA3).

Together, our single‐nuclei multiomic profiling identified ACSS2 as a key regulator of gene expression in the hippocampal perforant path of mice injected with AD tau. Loss of this enzyme resulted in exacerbated transcriptional dysregulation in GCs as well as CA3 and CA1 neurons, with increased ectopic expression of various genes, potentially due to loss of repressors as seen in the bulk RNA‐seq. The observed loss of synaptic genes might contribute to the increased amplitude of learning impairments.

### 3.5 AD tau–injected ACSS2 KO mice exhibit transcriptional dysregulation and depletion of Cajal–Retzius neurons

**FIGURE 5:**
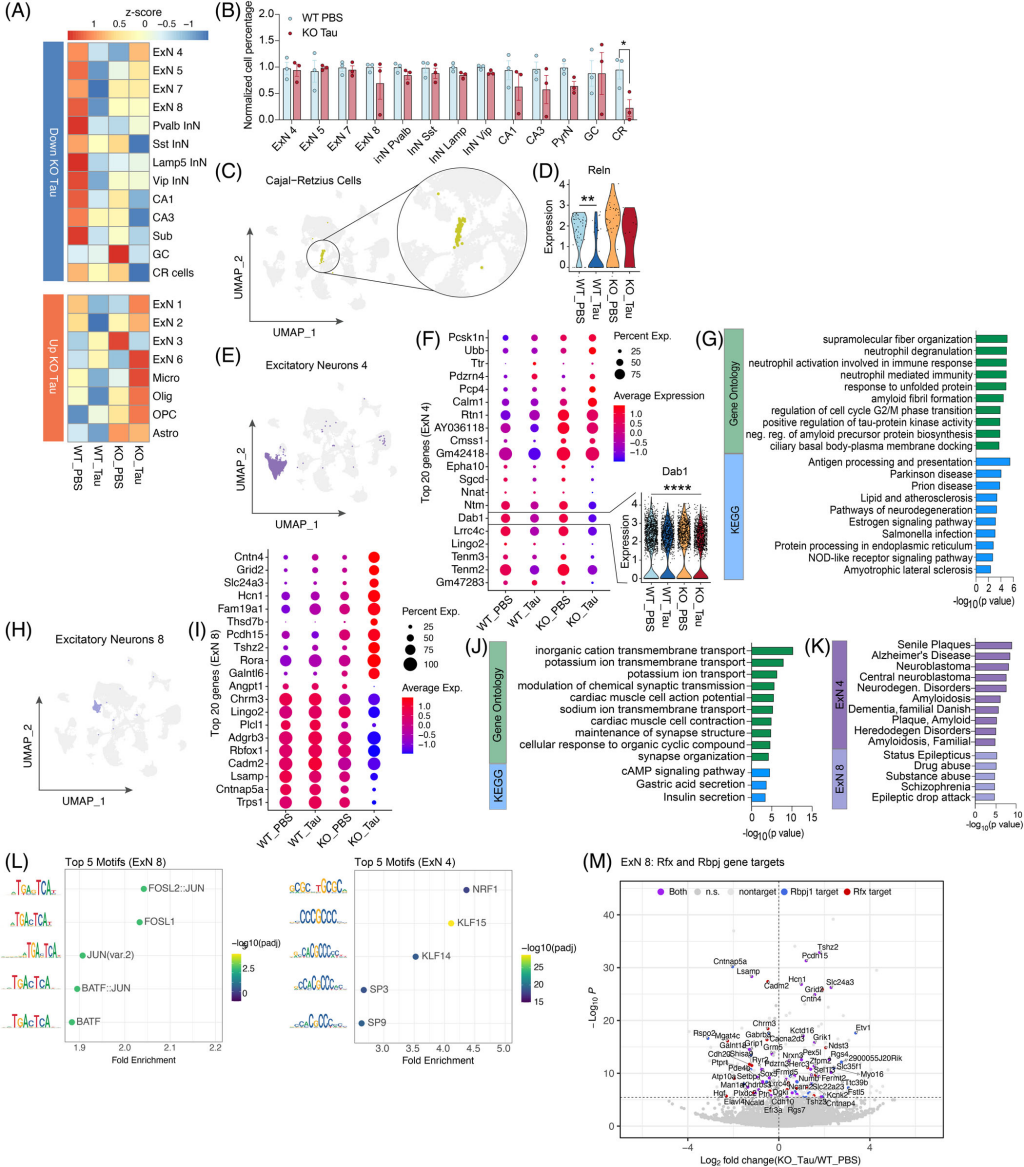
AD tau–injected ACSS2 KO mice exhibit dysregulation in other excitatory neuronal populations. A, Heatmap of cellular abundance across cell types. Clusters 22 to 24, likely dissection artefacts, removed prior to plotting. B, Barplot showing cell types from the top cluster of 5A, with all cell abundances normalized to WT PBS condition. Significance determined propeller (*n* = 3 per condition). C, Location of CR cells on the UMAP projection. D, Violin plot showing Reelin (Reln) expression in CR cells across all four conditions. E, Location of excitatory neuron 4 (ExN4) cluster on the UMAP projection. F, Dotplot of top 10 upregulated and downregulated genes between WT PBS and KO tau condition in the ExN4 cluster. Inset, Violin plot showing Dab1 expression in ExN4 across all four conditions. G, Top, Top 10 GO terms (*q* value < 0.1) for DEGs (*P*adj < 0.1) upregulated in KO tau. Bottom, Top 10 KEGG pathways (*q* value < 0.1) for DEGs (*P*adj < 0.1) upregulated in KO tau. H, Location of excitatory neuron 8 (ExN8) cluster on the UMAP projection. I, Dotplot of top 10 upregulated and downregulated genes between WT PBS and KO tau condition in the ExN8 cluster. J, top, All significant GO terms (*q* value < 0.1) for DEGs (*P*adj < 0.1) upregulated in KO tau. Bottom, All significant KEGG pathways (*q* value < 0.1) for DEGs (*P*adj < 0.1) upregulated in KO tau. K, top, Top 10 disease terms (*q* value < 0.1) in ExN4. Bottom, All significant disease terms in ExN8 for DEGs (*P*adj < 0.1) upregulated in KO tau (DisgeNET). L, Motif analysis of differentially accessible regions (DARs) in ExN8 (left) and ExN4 (right). M, Volcano plot showing DEGs in ExN8 between PBS‐injected WT mice (WT Ctrl) and tau‐injected ACSS2 KO mice (KO tau; *P*adj < 0.1). Significant Rfx targets shown in red, Rbpj1 in blue, and genes targeted by both in purple. ACSS2, acetyl‐CoA synthetase 2; AD, Alzheimer's disease; CR, Cajal–Retzius; Dab1, Disabled‐1; DEG, differentially expressed gene; GO, Gene Ontology; KEGG, Kyoto Encyclopedia of Genes and Genomes; KO, knockout; PBS, phosphate‐buffered saline; UMAP, Uniform Manifold Approximation and Projection; WT, wild type.

We next examined how cell populations shifted across tau injection or ACSS2 KO (Figure 5A). We contrasted KO tau cluster populations against WT Ctrl using a linear modeling–based method to test for alterations in cell number. No clusters showed significant increases in KO tau mice (Figure S6A in supporting information). The cell type that showed the only significant degree of loss was a small neuronal population, which we identified as Cajal–Retzius (CR) cells (Figure 5B, right) based on tandem expression of Slc17a7/VGLUT1, an excitatory neuronal marker, and Reelin (*Reln*), a secreted signaling protein critical for neuronal migration and axon guidance,^83^ as well as their small number (Figures 3, 5, cluster 21). CR cells were first identified as a transient neuronal population that governs neuronal migration during embryonic/early post‐natal cortical patterning.^84^ However, a small population persists in adult mice and humans, and the highest numbers are found in the molecular layer of the dentate gyrus,^85^ regions that show the highest degree of tau pathology in our model (Figure 1A). In our identified CR population, in WT mice, tau injection led to a significant decrease in Reln expression (*P*adj < 0.01; Figure 5D), and a significant reduction of CR cells in KO mice (Figure 5B), indicating a particular susceptibility to the double‐hit in this small neuronal population.

Disabled‐1 (Dab1) is the major intercellular mediator of Reln signaling in target cells, and is downregulated in certain cell populations in AD.^86^ We therefore examined all cell clusters for reductions in Dab1 expression levels, and identified a subpopulation of excitatory neurons (ExN4, identified by high expression of Hs3st4 and Cdh18 [Figure S3C]) as having significantly downregulated Dab1 (*P*adj < 3.42 × 10^−7^) expression in KO tau mice compared to WT controls (Figure 5E,F). In contrast, genes upregulated with KO tau in these ExN4 neurons fit into GO terms associated with inflammation, endoplasmic reticulum stress, and Aβ response, and multiple neurogenerative KEGG pathways (Figure 5G). GO terms associated with axonogenesis predominated in the WT PBS condition for this cluster (Figure S6B), and, as in the GO categories associated with CA3 PCs, the top KEGG pathway was protein kinase B (PKB) activation (Figure S6C).

We then investigated another excitatory neuronal cluster (ExN8) with the highest amount of dysregulation specific to the KO tau condition (Figure 5H). This cluster is identified by high expression of *Zfp804b* and Ntng1 (Figure S3c). Similar to the bulk RNA‐seq data, this population showed a drastic expansion of dysregulated genes only upon combined ACSS2 loss and tau exposure (Figures 3E and 5I, Figure S3H). KO tau–biased genes indicated a hyperexcitable phenotype, with ontologies and pathways associated with elevated glutaminergic synaptic transmission (Figure 5J). Increased neuronal excitability is characteristic of early neurodegeneration,^87^ and increased synaptic activity has been recorded in murine neurons proximal to Aβ deposits^88^ and with overexpression of pathological human tau variants.^89^, ^90^ Once again, PKB activation emerged as the top GO category in this cluster (Figure S6D), indicating a shared mechanism across excitatory cell types.

DisgeNET analysis of upregulated genes in each excitatory neuronal cluster underlined these observations, with KO tau–biased genes in ExN4 mapping to neurodegenerative diseases, and those in ExN8 to seizure disorders^91^ (Figure 5K). Motif analysis under differentially accessible snATAC‐seq peaks between WT PBS and KO tau in each cluster further corroborated these results, with ExN8 showing enrichment in AP‐1 motifs, including the activity‐dependent transcription factors FOSL1 and JUN,^92^ and ExN4 KLF‐ and SP‐family motifs, which are involved in the oxidative stress response in neurons,^93^ and NRF1, which regulates multiple aspects of the unfolded protein response^94^ (Figure 5L).

In contrast to the bulk analyses (Figure 2E–J), we observed no downregulation of Rfx family members (Rfx3/5/7) and Rbpj1, likely due to low transcript abundance, and no motif enrichment within DARs in any of the single‐nuclei clusters. Nevertheless, Rfx and Rbpj1 target genes (FDR < 1.0 × 10^−10^) were strongly dysregulated in several cell types including the hippocampal perforant pathway (CA1, CA3, and GC) as well as in ExN2 and ExN8, key excitatory neuronal populations that were most impacted in the double‐hit KO tau mice. In these nuclei, Rfx and Rbpj1 target genes showed significant overlaps (FDR < 1.0 × 10^−10^) with DEGs from the WT PBS versus KO tau comparison (Figure S6E). For both TF families, we observed the most significant overlaps with DEGs in ExN8 (Figure S6F) and in GC (Figure S6G). In ExN8, shared targets of Rfx and Rbpj1 were among the most significantly dysregulated genes (Figure 5M) including *Ncam2*, a target of AD‐related synaptic loss.^95^ Similarly, Rfx and Rbpj1 target genes showed marked dysregulation in GCs (Figure S6H). An analysis of Rfx and Rbpj motifs across all snATAC‐seq DARs showed tau‐induced gains in bulk H3K27ac signal at the nearest annotated gene; these gains were largely exacerbated in the KO mouse (Figure S6I). Strikingly, we found no overlap between Rfx and Rbpj1 targets and DEGs in inhibitory neurons and glia (Figure S6E), emphasizing the important role of these transcriptional regulators specifically in cell types most affected by the double‐hit of tau seeding and ACSS2 loss.

Together, the multiomic profiling provided converging evidence for a robust impact on multiple excitatory neuronal cell clusters of ACSS2 loss and AD tau seeding, emphasizing a potential key role of ACSS2 in this hippocampal cell type in tauopathy, orchestrating a cascade of direct and indirect downstream chromatin and transcriptional changes. In addition, overall, we found that KO tau mice exhibit a profound transcriptional dysregulation of distinct excitatory neuronal populations in the hippocampus. This included a loss of Reln signaling and a significant reduction in the number of Reln‐expressing CR cells. The loss of this developmentally important cell type has been documented in models of AD,^85^ and our findings strongly corroborate their potential key role in tauopathy, and exacerbation by loss of ACSS2.

### 3.6 Acetate‐enriched diet ameliorates AD tau–induced molecular changes and memory deficits dependent on ACSS2

**FIGURE 6:**
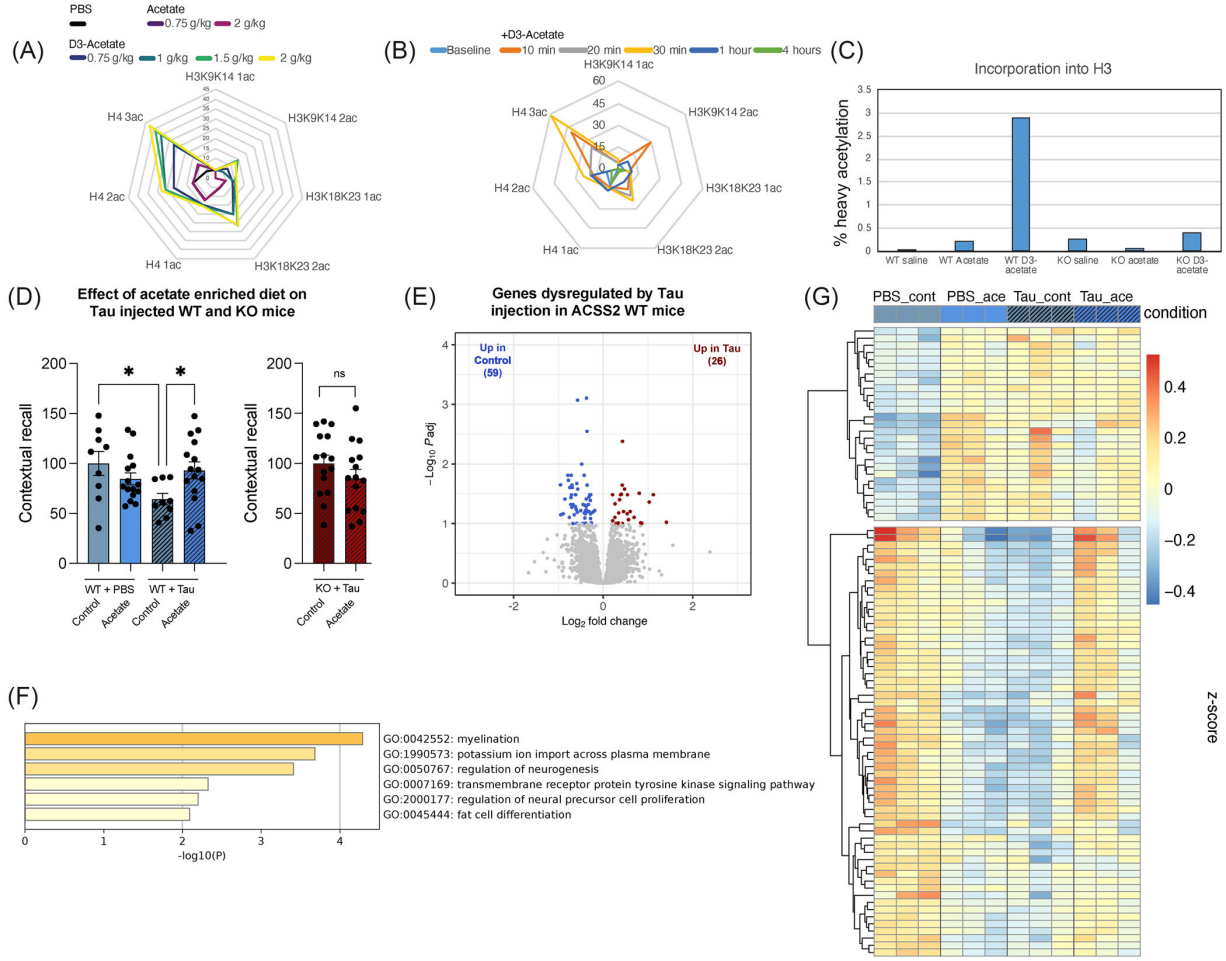
Acetate‐enriched diet ameliorates AD tau–induced molecular changes and memory deficits. A, Dose‐dependent heavy labeling of mouse hippocampal histone acetylation after d3‐acetate injection (*n* = 3). B, Temporal dynamics of heavy labeling of mouse hippocampal histone acetylation after d3‐acetate injection (*n* = 3). C, Heavy labeling of histone acetylation in WT but not ACSS2 KO mice after d3‐acetate injection. D, Dietary supplementation of acetate increases freezing behavior during recall of contextual fear memories in WT mice (Student *t*‐test, *t*_22_ = 2.397, *P* = 0.0254), but not ACSS2 KO mice (WT PBS *n* = 9; WT PBS+Ac *n* = 14; WT Tau *n* = 9; WT Tau+Ac *n* = 15; KO PBS *n* = 15; KO PBS+Ac *n* = 15). E, Volcano plot showing genes differentially regulated in tau‐injected WT mice compared to PBS controls (*n* = 3 per condition; *P*adj < 0.1). F, GO analysis of genes downregulated in tau‐injected WT mice. G, Heatmap of top 87 differentially regulated genes in tau‐injected WT mice compared to PBS controls, showing partial rescue of downregulated transcripts with acetate supplementation. ACSS2, acetyl‐CoA synthetase 2; AD, Alzheimer's disease; GO, Gene Ontology; KO, knockout; PBS, phosphate‐buffered saline; WT, wild type.

Our results underline a potentially key role of metabolic–epigenetic interactions, given the exacerbation of memory deficits in the mouse AD tau by ACSS2 KO. We therefore reasoned that increasing the levels of   acetate, the substrate of ACSS2, might have beneficial effects in our tauopathy model. Acetate supplementation in primary hippocampal neurons drives genes of learning and memory and promotes cognitive function in a developmental mouse model,^96^ enhances spatial learning in WT mice,^97^ and acetate gavage can promote memory in the 5xFAD mouse.^25^ To investigate and define mechanisms of acetate supplementation in modulation of gene function for learning and memory in normal mice, we first determined whether exogenous acetate is directly incorporated into hippocampal histone acetylation, using heavy labeled acetate. Mice were injected with d3‐acetate intraperitoneally (i.p.), and heavy labeling of acetylated histones was measured at different time points using stable isotope labeling MS. We observed a rapid, transient (peak labeling at 30 minutes), and dose‐dependent deposition of acetate on hippocampal histones (Figure 6A, B). Importantly, heavy label incorporation was nearly completely dependent on ACSS2, demonstrated by absence of heavy label incorporation into hippocampal histones in ACSS2 KO mice (Figure 6C). Hence, exogenous acetate can be directly incorporated into histone acetylation, and this requires ACSS2.

We then investigated whether supplying exogenous acetate can reverse learning and memory impairments in the context of AD tau injection. WT mice were injected with 1 µg AD tau in the dorsal hippocampus and underwent FC 6 months post‐injection. We found that acute exposure to acetate (1.5 g/kg i.p. prior to acquisition) did not rescue decreased freezing in this model (Figure S7A in supporting information); thus, we hypothesized that chronic administration of acetate might be required to reverse long‐lasting effects of AD tau seeding. To achieve chronic acetate administration, we maintained mice on a special diet enriched in acetate 5% (w/w) continuously through 6 months after AD tau injection. As previously reported,^98^ mice administered an acetate diet had slightly reduced body weight compared to controls maintained on a calorically equivalent diet (Student *t* test, *t*_46_ = 2.236, *P* = 0.0302; Figure S7B,C); however, there was no apparent sickness, or measured anxiety, or impairment of general locomotion in open field testing (Figure S7D, E). Chronic acetate supplementation did not alter freezing behavior in mice that were not AD tau injected (Student *t* test, *t*_22_ = 1.276, *P* = 0.2153; Figure 6D, left). However, in AD tau–injected mice, the acetate diet improved contextual recall, evidenced by nearly 50% increased freezing behavior (Student *t* test, *t*_22_ = 2.397, *P* = 0.0254; Figure 6D, middle). Notably, the acetate‐ enriched diet did not improve recall in ACSS2 KO mice, indicating that ACSS2 is required this effect required ACSS2 as amelioration was observed in WT tau–injected mice, whereas the acetate‐enriched diet did not improve recall of fear‐associated contextual cues in ACSS2 KO mice (Student *t* test, *t*_28_ = 1.238, *P* = 0.2261; Figure 6D, right).

Finally, we examined the transcriptional landscape underlying the acetate‐mediated alleviation of tau‐related deficits. WT mice were seeded with 1 µg AD tau and maintained for 6 months on an acetate‐rich or control diet. Control mice were sham injected with PBS. We performed bulk RNA‐seq on the dorsal hippocampus of representative animals from each group (*n* = 3), selecting the side where AD tau had been seeded 6 months prior. Consistent with previous results (Figure 2A), we found that tau injection led to a modest number of DEGs in mice fed standard chow (Figure 6E). We noted the GO terms for genes reduced by tau injection included neurogenesis and myelination (Figure 6F), impairments of which are features of human AD.^99^, ^100^ Strikingly, of the 56 genes downregulated by tau injection, all showed recovery with acetate supplementation (Figure 6G, bottom), providing strong evidence that acetate supplementation can enhance long‐term memory and facilitate neuronal resilience and recovery, manifested in improved gene expression profile.

Together, these data indicate that boosting ACSS2‐dependent histone acetylation by dietary supplementation of acetate rescues long‐term memory in a mouse model of tauopathy via altered histone acetylation and gene expression, while exhibiting no discernible side effects.

## 4 DISCUSSION

We establish ACSS2 as a novel modulator of tau‐related phenotypes in a human tau seeding mouse model. We report that loss of ACSS2 exacerbates memory impairments in this model, and further impacts transcriptional and epigenetic dysregulation without worsening tau pathology (Figure 1). Multiomic characterization of the underlying chromatin and gene expression changes using single‐nuclei approaches identified populations of excitatory neurons, including those along the perforant path and CR cells, as critical hippocampal cell populations affected by AD tau seeding (Figure 4). We find that loss of ACSS2 in the context of AD tau results in complex cellular and genomic alterations in these neurons, including ectopic upregulation of gene expression programs leading to loss of cell identity and increased disease risk. Further, we find that dietary supplementation of acetate, the substrate of ACSS2, rescues memory (Figure 6). This effect is mediated by ACSS2‐dependent incorporation of acetate directly into hippocampal histone acetylation, which induces gene expression programs that facilitate learning (Figure 6).

Tau seeding has been primarily used to study mechanisms that govern the assembly of tau into filamentous inclusions, as well as the spreading of tau pathology across brain regions and differences between distinct conformers.^29^, ^55^ Notably, here we show that the molecular responses to AD tau injection reflect some of the molecular characteristics of human AD (Figure 2 and Figure S2). Specifically, histone acetylation and transcriptional changes induced by AD tau injection mirror those previously reported in the brain of AD patients.^8^, ^9^ Genes associated with the top AD tau–induced H3K27ac peaks show a striking enrichment in genes dysregulated in human AD;^101^, ^102^ several DEGs are linked to disease risk, neuronal death, and memory loss.^103^ Importantly, these findings support AD tau injection as an informative mouse model of tauopathy that, in addition to tau pathology, also recapitulates the epigenetic and transcriptional changes reflective of the brain of human AD patients. Nevertheless, other key components of human AD (such as amyloid deposition, innate immune activation, blood–brain barrier dysfunction, chronic glial reactivity, and others) are not captured by this model, and the biochemical properties, post‐translational modifications, and conformational states of exogenous tau may differ substantially from those in sporadic AD brains. Thus, future work using additional mouse models capturing a broader spectrum of AD pathophysiology will be critically important to corroborate our findings.

One advantage of this model is that AD tau seeding can be done in any genetically engineered mouse lines enabling broad testing of combinatorial effects on AD phenotypes. By injecting AD tau into ACSS2 KO mice, we show that ACSS2 regulates tau‐induced epigenetic and gene expression changes separately from hippocampal tau pathology. This is important as previous and current therapeutic approaches focusing on tau filaments and amyloid plaque formation have proven largely unsuccessful, necessitating the identification and targeting of alternative disease mechanisms. Interestingly, upregulation of ACSS2 ameliorates tau pathology in other models of tauopathy.^104^ While some of our findings, particularly the higher number of DEGs in bulk RNA‐seq, point to potential synergy between ACSS2 loss and tau pathology, it remains unclear whether our double‐hit model captures a true mechanistic interaction or the sum of two parallel hits. Thus, a systematic pathological, molecular, and behavioral comparison of tau seeding and other widely used AD models in the context of ACSS2 KO is warranted to guide future efforts focusing on distinct aspects of the disease.

Despite the emergence of histone acetylation as a key epigenetic mechanism contributing to AD, developing therapeutic approaches targeting this pathway has proven challenging due to lack of specificity and toxic side effects. Our results underscore ACSS2 as a key molecular target for future therapy development. Upregulation of ACSS2 and acetate gavage have been shown to improve learning and memory for the Morris water maze in an APP mouse model.^25^ Upregulation of ACSS2 alone ameliorates not only long‐term memory, but also tau pathology in PS19 mice.^104^ Here we show that dietary supplementation of acetate ameliorates memory impairments in an AD tau–injected mouse—an effect that is dependent on ACSS2‐mediated histone acetylation in the hippocampus. Consistent with a key role for ACSS2, inhibition of ACSS2 by genetic deletion severely exacerbates learning deficits in mice injected with AD tau. Importantly, these manipulations had no effect on short‐term memory, anxiety‐like behavior, or general locomotion, and thus hold promise for increased specificity and lower toxicity in a clinical setting. Further, dietary supplementation of acetate is entirely non‐invasive and would likely pose limited compliance issues during chronic administration.

Our multiomic approach identifies potential new mechanisms underlying tauopathy. Previously, we showed that ACSS2 maintains nuclear pools of acetyl‐CoA in the hippocampus, thus fueling histone acetylation and gene expression required for memory formation.^21^ Here, we find that the loss of this key neuroprotective enzyme leads to the worsening of both molecular and behavioral phenotypes. Reduced acetyl‐CoA and histone acetylation might thus be key mechanisms of disease etiology, and similar epigenetic changes have been observed in human patients.^8^, ^9^ Further, we identify the loss of transcriptional repressor RFX3 as a novel potential disease mechanism. We find that RFX3 expression is decreased in our mouse model, which may enable the aberrant expression of target genes that are silenced in the healthy brain. The increased expression of RFX3 target genes is likely driven by a transcriptional activator(s), as we found enrichment of RFX3 motifs in loci that gained H3K27 acetylation (hence likely became more accessible) in KO tau mice. Within our snRNA‐seq, we identified enrichments of differentially regulated RFX targets in several neuronal populations, most notably GCs and a subset of ExN8. Future identification of relevant transcriptional activators is of great interest. While the precise transcriptional regulation remains to be elucidated, including further characterization of Rfx3 binding across disease states, the ectopic expression of RFX3 target genes unrelated to neuronal function could be a key early molecular step leading to impaired plasticity and cognition.

Our snRNA‐seq and ATAC‐seq data highlight key hippocampal circuits and neuronal populations that are particularly susceptible to the double hit of tau seeding and ACSS2 loss. In particular, ExN8 along the perforant path—including GCs and PCs of the CA3 and CA1 subfields—showed some of the largest alterations in transcription, chromatin accessibility, and cell number in the double‐hit condition. Synaptic genes were the most consistently altered group of genes across all three clusters, with Sema3c, a secreted axon guidance factor,^105^ and Grik1, an ionotropic glutamate receptor,^106^ showing uniformly large downregulation in the KO tau condition. Loss of synaptic function along the perforant path is a hallmark of AD and is correlated with cognitive impairment.^107^ Of the three clusters within the perforant path, GCs exhibited the largest dysregulation in transcription and chromatin accessibility. This level of dysregulation could be related to the proximity of GCs to phosphorylated tau aggregates in the dentate gyrus. However, the identities of these dysregulated genes were primarily synaptic, and reflected few changes directly associated with neurodegenerative processes. While the CA3 and CA1 subfields exhibited less intense tau accumulation, the genes upregulated in the KO tau condition strongly associated with neurodegenerative profiles. In addition, while GCs showed no significant decrease in cell number in the double‐hit condition, both CA3 and CA1 exhibited significant losses, preceded by smaller reductions in the WT tau condition alone. These findings suggest that ACSS2 plays an important neuroprotective role in the hippocampus, loss of which exacerbates epigenome erosion and transcriptional dysregulation, resulting in cell identity loss of key hippocampal populations and more severe cognitive impairments.

The increased transcriptional dysregulation in critical neuronal populations distal to the site of the most intense tau aggregation may be driven by loss of homeostatic signaling pathways. We observed strong concordance in reduction of PKB activation‐related pathways across multiple affected cell types (CA3, ExN4, and ExN8), agreeing with reports that PKB activation enhances axonal integrity and neuronal survival in neurodegenerative models.^79^, ^80^ We also found that CR cells, a small subpopulation of hippocampal excitatory neurons, exhibited the largest degree of loss in the double‐hit KO tau condition. CR cells are glutamatergic neurons in the cortex and hippocampus and are primarily studied in the context of brain development, although they are linked to learning and memory in adult animals.^108^, ^109^ Notably, CR cells are a major source of Reln, which regulates axon guidance and synaptic plasticity in the developing and adult brain.^110^ Reln signaling is heavily disrupted in AD,^86^ and reduced in both *post mortem* human AD brains and in mouse models. Loss of this pathway reduces synaptic structure and glial ensheathment,^111^ and leads to increased accumulation of pathological phospho‐tau and Aβ aggregates.^112^, ^113^, ^114^ Further, loss of CR cells at various developmental time points occurs in a transgenic mouse model of AD.^85^, ^115^ Intriguingly, the number of CR cells shows remarkable plasticity and increases upon environmental enrichment,^116^ which has beneficial effects on cognition. Whether dietary supplementation of acetate exerts its therapeutic effects by acting on CR cells will be an important area for future investigation.

Together, our findings establish AD tau injection as a mouse model of tauopathy that partially reflects molecular changes observed in human patients. We show that epigenetic and transcriptional dysregulation is exacerbated after ACSS2 KO independent from tau pathology, and we identify key neuronal populations as contributors to the molecular re‐configuration of the tau‐seeded hippocampus. Further, genetic deletion of ACSS2 significantly worsens long‐term memory in the context of tau injection. This finding underscores an important neuroprotective role of ACSS2‐mediated histone acetylation in the hippocampus. Indeed, dietary supplementation of acetate, the substrate of ACSS2, rescues learning and memory in an ACSS2‐dependent manner. Overall, this work establishes ACSS2 and related epigenetic and transcriptional dysregulation and resulting cell identity loss as a novel potential mechanism of impaired cognition in a mouse model of tauopathy, with the potential to inform the development of future therapies.

## CONFLICT OF INTEREST STATEMENT

Shelley Berger is the cofounder of EpiVario, Inc. All other authors declare that they have no competing interests. Author disclosures are available in the supporting information.

## CONSENT STATEMENT

Consent for data obtained in this manuscript was not necessary, as no human subjects were involved.

## Supporting information



Supporting Information

Supporting Information

Supporting Information

## Data Availability

All bulk RNA‐seq, ChIP‐seq, snRNA‐seq, and scATAC‐seq data are available at GEO superseries GSE294261. Custom scripts and pipelines are available upon request.
