# Supplementary material for: Multiomic single nuclei profiling the mouse hippocampus reveals that ACSS2 confers neuronal resilience to tauopathy
Source: Alzheimers Dement. 2026 Feb 13;22(2):e70998. doi: 10.1002/alz.70998 (PMC12902802; doi:10.1002/alz.70998)
Supplement: Supplementary file 2 — Supporting Information [file ALZ-22-e70998-s003.pdf]

Extended Data Fig 1. Neither AD-Tau injection nor ACSS2 KO impacts baseline behavior.

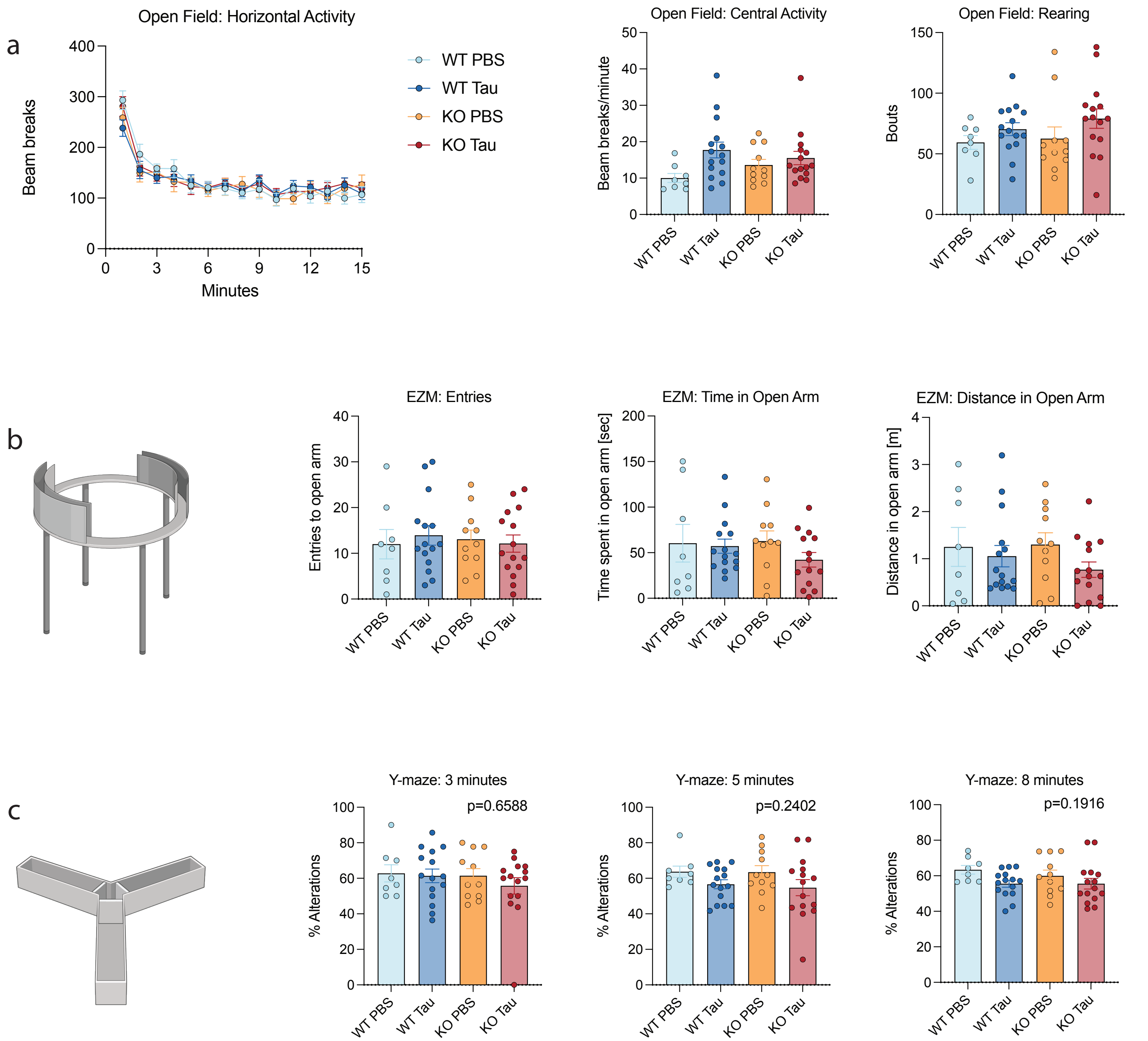

Supplemental Figure 1. Neither AD-Tau injection nor ACSS2 KO impacts baseline behavior.

A. Open field (OF) parameters across Tau-injected WT (WT-Tau) and ACSS2 KO (KO Tau) mice and PBS controls (WT PBS and KO PBS). (left) total movement in the open field over the 15 minute assay period. (center) activity in the center of the arena. (right) Bouts of rearing behavior over the 15 minute assay period. (WT PBS: n=8; WT Tau: n= 14; KO PBS: n=11; KO Tau: n=14). Bar plots represent mean ± SEM, with individual points representing single animals. **B.** Measured Elevated Zero Maze (EZM) parameters across Tau-injected WT (WT-Tau) and ACSS2 KO (KO Tau) mice and PBS controls (WT PBS and KO PBS). (absolute left) Schematic of the elevated zero maze.(left) Entries made to open arm over the 5 minute assay period. (center) Time (in seconds) spent in open arms of the arena. (right) Distance traversed (meters [m]) in open arms of the arena. **C.** Percent alternations in Y-maze assay binned by (left) first 3 minutes; (center) first 5 minutes; (right) total 8 minute assay period.

Extended Data Fig 2. AD-Tau injection in mouse hippocampus recapitulates human AD epigenetic changes.

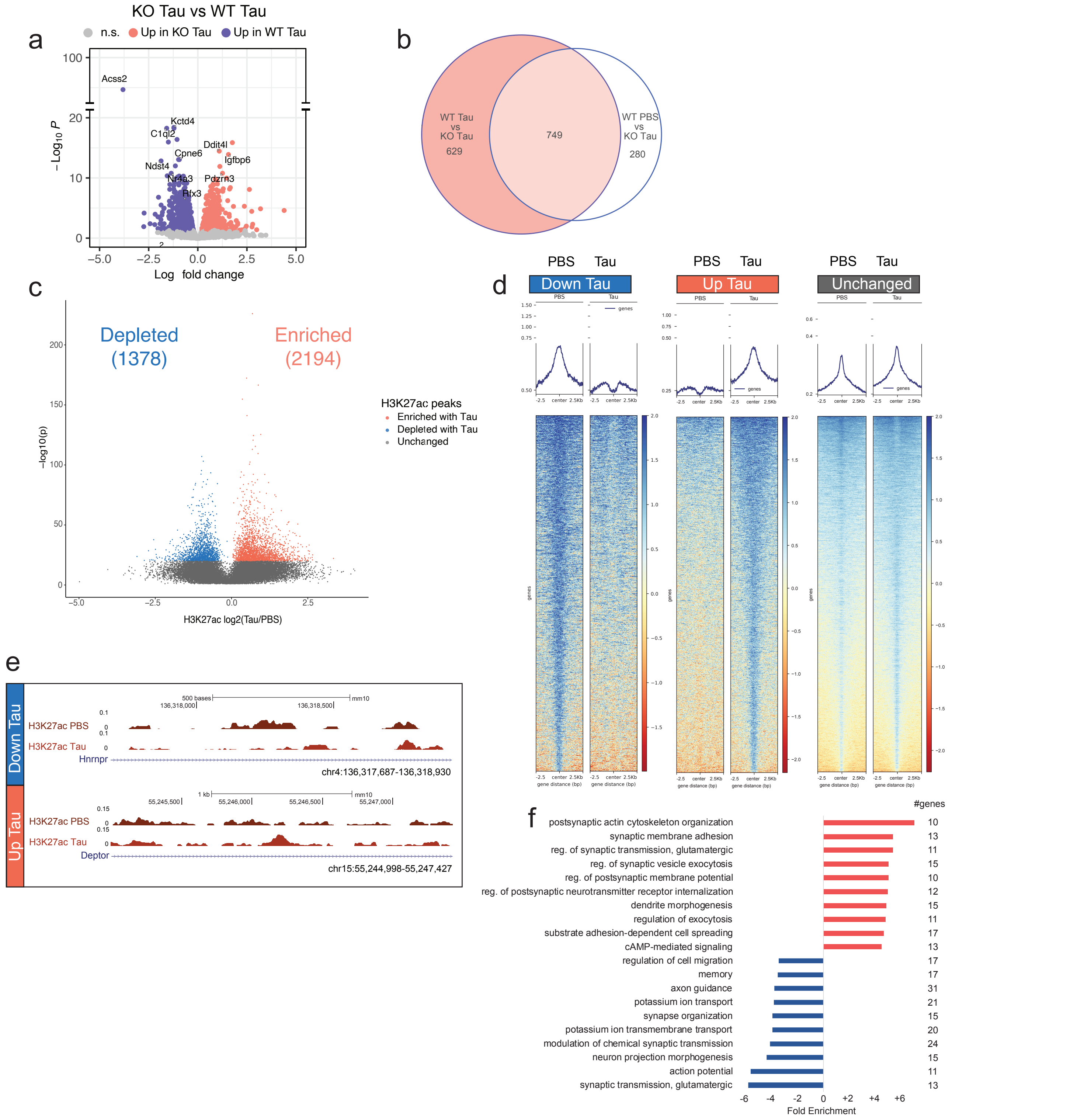

Supplemental Figure 2. AD-Tau injection in mouse hippocampus recapitulates AD-related epigenetic changes.

A. A volcano plot contrasting the hippocampal transcriptome of Tau-injected ACSS2 KO vs WT mice via RNAseq. **B.** Venn Diagram showing overlap of DEGs between WT PBS vs KO Tau and WT Tau vs KO Tau (hypergeometric test: p = 0). **C.** A volcano plot contrasting H3K27ac peaks in Tau-injected and PBS control. Dots represent peaks filtered to the most significant peak per gene (scored by THOR - adjusted p < 1E-20 and log2(Tau/PBS) > 0) (Tau: N = 2,194; PBS: N=1378; Unchanged: N=16,226). **D.** Heatmaps showing 5kb windows around all H3K27ac peaks in Tau or PBS, separated by Tau-specific, PBS-specific, or Common (N = 15,481 PBS-specific, 8,856 Tau-specific, and 27,540 common peaks). **E.** Overlapping H3K27ac enrichment at the Hnrnpr and Deptor loci in the hippocampus of AD-Tau injected mice vs PBS controls (top) **F.** Gene Ontology analysis of H3K27ac peaks induced by AD-Tau injection in mouse hippocampus.

Extended Data Fig 3. Single nuclei profiling of gene expression and chromatin accessibility of AD-Tau-injected ACSS2 KO mice

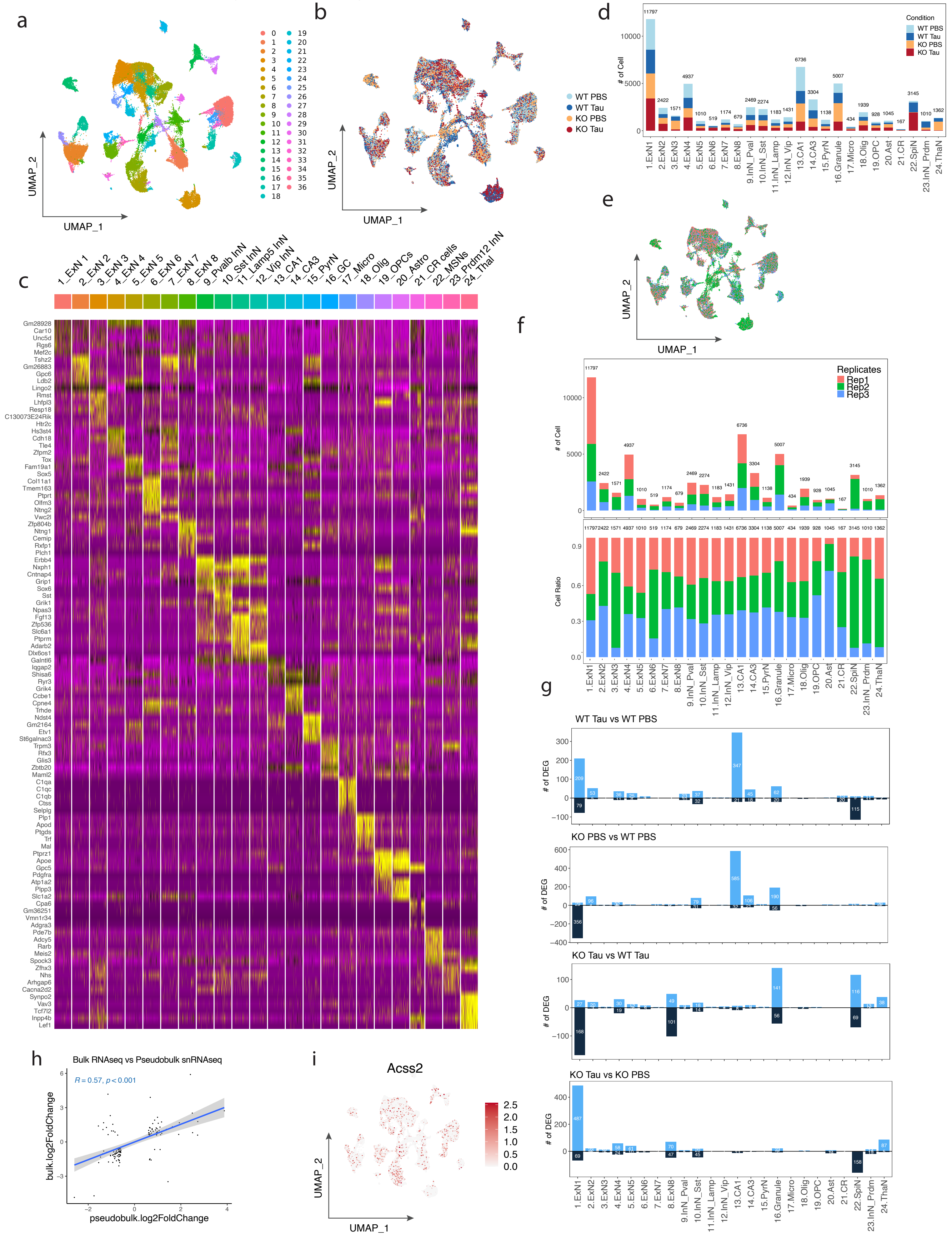

Supplemental Figure 3. Single nuclei profiling of gene expression and chromatin accessibility of AD-Tau-injected ACSS2 KO mice

A. UMAP embedding of the snRNAseq dataset, featuring all 36 clusters originally determined by Seurat. **B.** UMAP embedding of the snRNAseq dataset, colored by condition. **C.** Heatmap of the top 5 genes per final annotated cluster. **D.** Cluster-specific ratios in cell number across conditions (genotype, injection). Column sizes scaled to number of cells in each cluster. Total number of cells per cluster indicated above each column. **E.** UMAP embedding of the snRNAseq dataset, colored by replicate. **F.** Cluster-specific ratios in cell numbers between replicates. Column sizes scaled to number of cells in each cluster (left) and as normalized cell ratio (right). Total number of cells per cluster indicated above each column. **G.** Differentially expressed genes across all clusters for remaining contrasts. **H**. Scatterplot comparing log2FoldChange inbulk RNA-seq vs pseudobulk snRNA-seq using genes significant in both (abs(FC)>1.5& pvalue<0.05, total N=117). **I.** UMAP embedding of snRNAseqdataset, showing Acss2 transcript levels within single cells.

Extended Data Fig 4. Single nuclei profiling of gene expression and chromatin accessibility of AD-Tau-injected ACSS2 KO mice

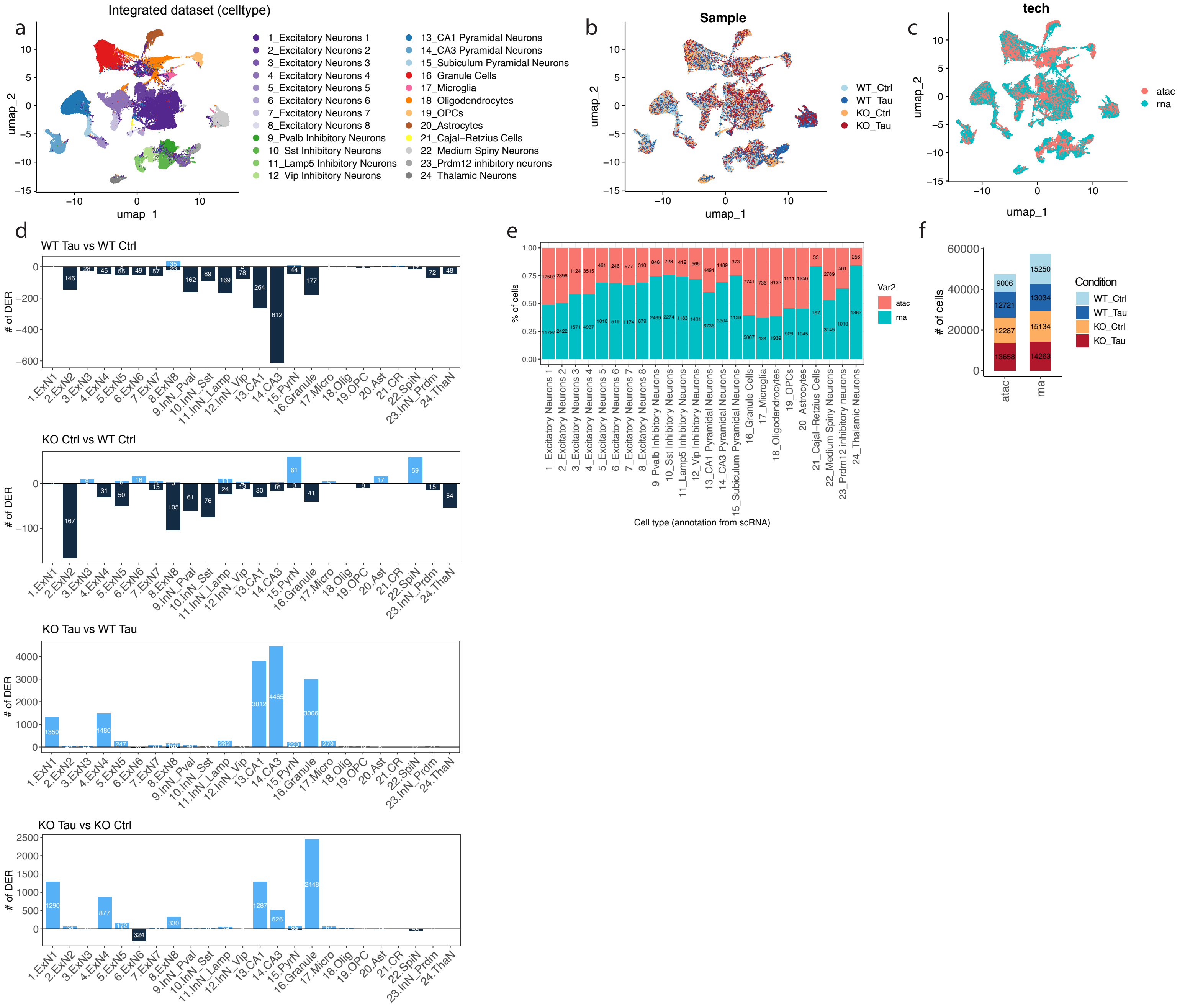

Supplemental Figure 4. Single nuclei profiling of gene expression and chromatin accessibility of AD-Tau-injected ACSS2 KO mice

A. UMAP of integrated 10x multiome dataset, including scRNA and ATACseq, colored by all annotated clusters. B. Data from A, colored by condition. C. Data from A, colored by technical source of cells (ATAC or RNA). D. Differentially accessible regions across all clusters for remaining contrasts. E. Barplot showing percentages of cells called by either method within each annotated cluster. F. Barplot showing percentage of cells recovered across all four conditions by method.

Extended Data Fig 5.
AD-Tau-injected ACSS2 KO mice exhibit transcriptional dysregulation of hippocampal circuitry

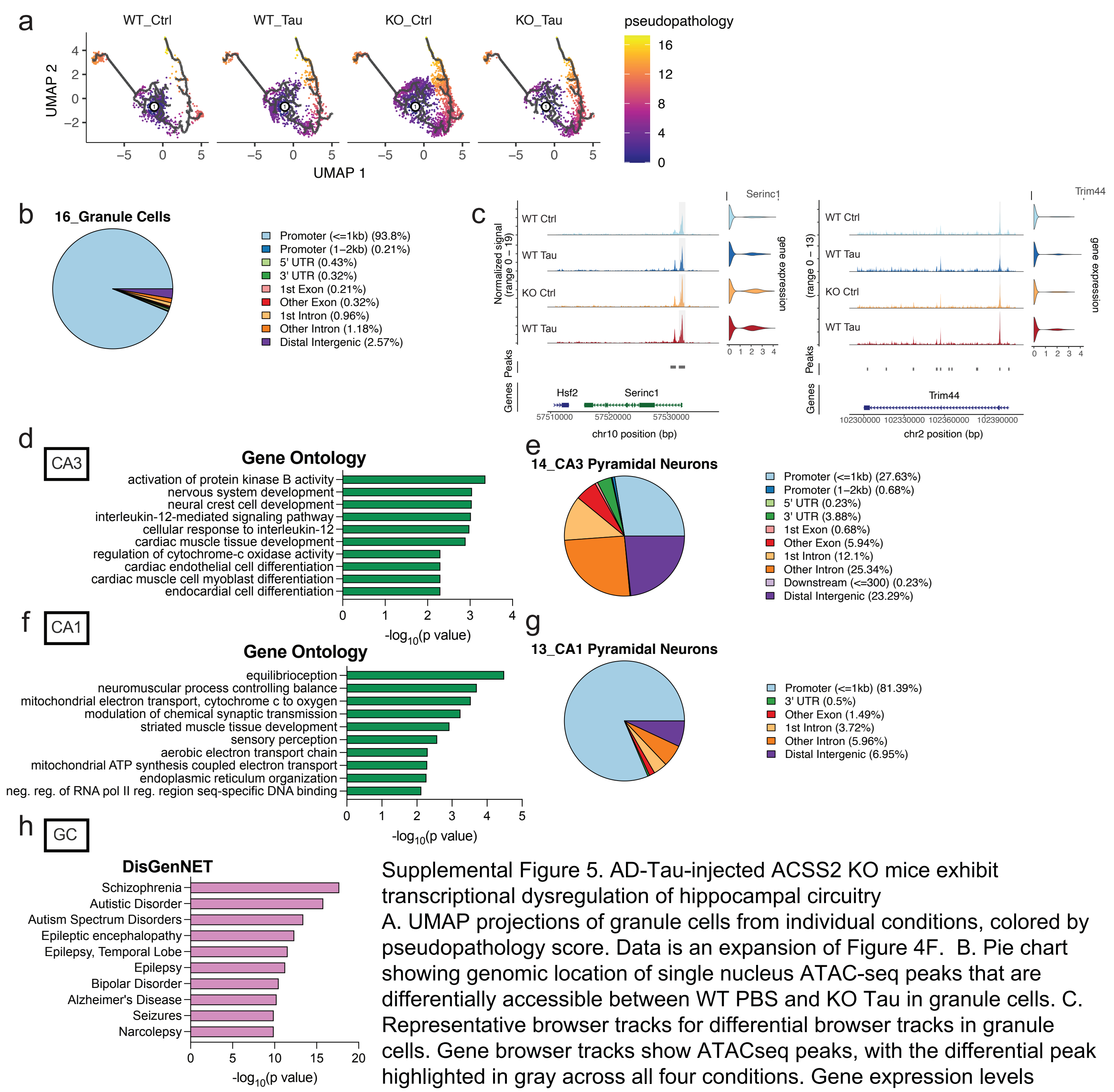

Supplemental Figure 5. AD-Tau-injected ACSS2 KO mice exhibit transcriptional dysregulation of hippocampal circuitry
A. UMAP projections of granule cells from individual conditions, colored by pseudopathology score. Data is an expansion of Figure 4F. B. Pie chart showing genomic location of single nucleus ATAC-seq peaks that are differentially accessible between WT PBS and KO Tau in granule cells. C. Representative browser tracks for differential browser tracks in granule cells. Gene browser tracks show ATACseq peaks, with the differential peak highlighted in gray across all four conditions. Gene expression levels shown in violin plots to the right. D. Top 10 Gene ontology terms (qvalue <0.1) for differentially expressed genes (padj < 0.1) upregulated in WT PBS in CA3 pyramidal cells (PCs). E. Pie chart showing genomic location of single nucleus ATAC-seq peaks that are differentially accessible between WT PBS and KO Tau in CA3 PCs. F. Top 10 Gene ontology terms (qvalue <0.1) for differentially expressed genes (padj < 0.1) upregulated in WT PBS in CA1 pyramidal cells (PCs). G. Pie chart showing genomic location of single nucleus ATAC-seq peaks that are differentially accessible between WT PBS and KO Tau in CA1 PCs. H. Top 10 disease terms (qvalue <0.1) for differentially expressed genes (padj < 0.1) upregulated in KO Tau Granule Cells (DisgeNET).

Extended Data Fig 6.

AD-Tau-injected ACSS2 KO mice exhibit dysregulation in other excitatory neuronal populations.

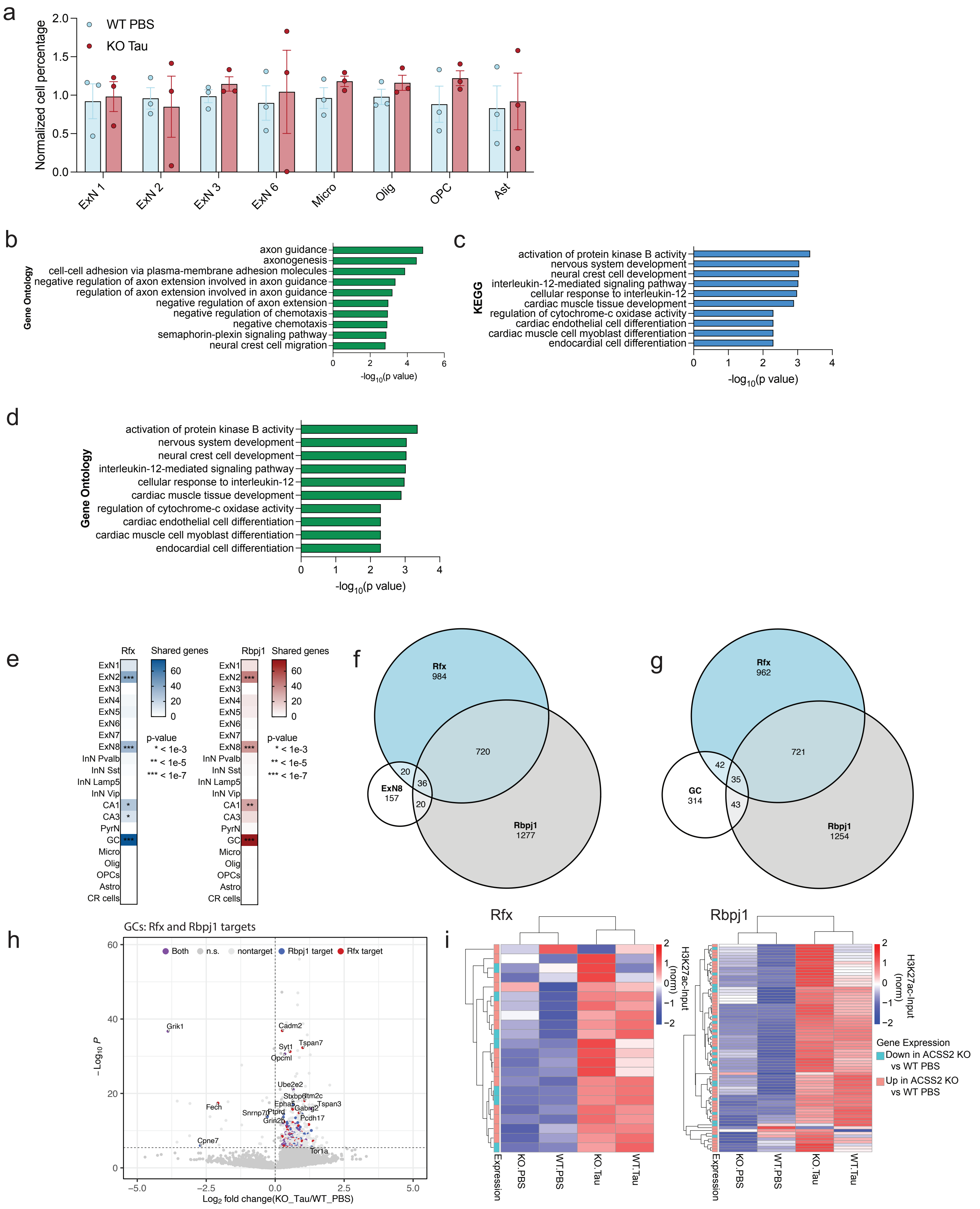

Supplemental Figure 6. AD-Tau-injected ACSS2 KO mice exhibit dysregulation in other excitatory neuronal populations.

A. Barplot showing cell types from the bottom cluster of Figure 5A, with all cell abundances normalized to WT PBS condition. Significance determined via propeller.(n=3 per condition) **B.** Top 10 Gene ontology terms (qvalue <0.1) for differentially expressed genes (padj < 0.1) upregulated in WT PBS in Ex N 4. **C.** Top 10 KEGG pathways (qvalue <0.1) for differentially expressed genes (padj < 0.1) upregulated in WT PBS in Ex N 4. **D.** Top 10 Gene ontology terms (qvalue <0.1) for differentially expressed genes (padj < 0.1) upregulated in WT PBS in Ex N 8. **E.** Heatmap showing number of overlapping genes between cluster-specific DEGs (KO Tau vs WT PBS) and genes targeted by Rfx family members (left) and Rbpj1 (right). Significance determined via hypergeometric test. **F.** Venn diagram showing ExN8-specific DEGs overlapped with curated lists of Rfx and Rbpj1 targets. **G.** Venn diagram showing GC-specific DEGs overlapped with curated lists of Rfx and Rbpj1 targets. **H.** Volcano plot showing differentially expressed genes in GCs between PBS-injected WT mice (WT Ctrl) and Tau-injected ACSS2KO mice (KO Tau) (padj < 0.1). Significant Rfx targets shown in red, Rbpj1 in blue, and genes targeted by both in purple. **I.** Heatmaps showing bulk log2(H3K27ac-Input) signal at genes proximal to merged scATAC-seq peaks featuring motifs for Rfx (left) and Rbpj1 (right).

Extended Data Fig 7. Effects of acetate supplementation on AD-Tau injected mice

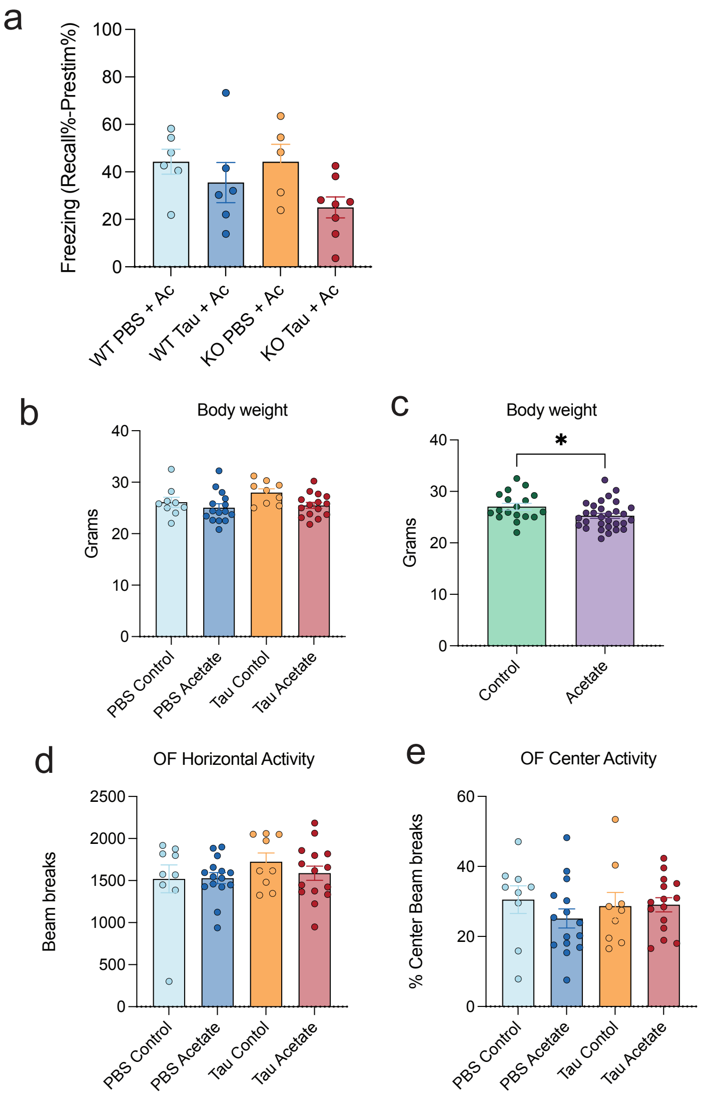

Supplemental Figure 7. Effects of acetate supplementation on AD-Tau injected mice

A. Freezing levels at 24h show no difference in fear conditioning recall between groups for Tau-injected WT and ACSS2 mice injected intraperitoneally with aceate (1.5 g/kg) prior to acquisition. B. Body weight of Tau- or PBS-injected WT mice maintained on high acetate diet compared to control chow. Data binned by individual group. C. Data presented in Figure B binned by diet composition alone. D. OF total movement in the open field arena over the 15 minute assay period. E. Activity in the center of the arena.
